# Expanding CIRdb, a comprehensive catalog of whole-exome sequencing data of Canary Islanders

**DOI:** 10.1016/j.isci.2026.117146

**Published:** 2026-08-10

**Authors:** Ana Díaz-de Usera, Luis A. Rubio-Rodríguez, Adrián Muñoz-Barrera, Jose M. Lorenzo-Salazar, Beatriz Guillen-Guio, David Jáspez, Almudena Corrales, Itahisa Marcelino-Rodríguez, María Del Cristo Rodríguez-Pérez, Antonio Cabrera-de León, Rafaela González-Montelongo, Raquel Cruz-Guerrero, Ángel Carracedo, Carlos Flores

**Affiliations:** 1 Genomics Division, Instituto Tecnológico y de Energías Renovables (ITER), 38600 Granadilla de Abona, Spain; 2 Division of Public Health and Epidemiology, School of Medical Sciences, University of Leicester, LE1 7HA Leicester, UK; 3 NIHR Leicester Biomedical Research Centre, LE3 9QP Leicester, UK; 4 CIBER de Enfermedades Respiratorias (CIBERES), Instituto de Salud Carlos III, 28029 Madrid, Spain; 5 Research Unit, Hospital Universitario Nuestra Señora de Candelaria, Instituto de Investigación Sanitaria de Canarias (IISC), 38010 Santa Cruz de Tenerife, Spain; 6 Área de Medicina Preventiva y Salud Pública, Universidad de La Laguna, 38200 San Cristóbal de La Laguna, Spain; 7 Instituto de Tecnologías Biomédicas, Universidad de La Laguna, 38200 San Cristóbal de La Laguna, Spain; 8 Centro de Investigación Biomédica en Red de Enfermedades Raras (CIBERER), Instituto de Salud Carlos III, 28029 Madrid, Spain; 9 Fundación Pública Galega de Medicina Xenómica, Sistema Galego de Saúde (SERGAS), 15706 Santiago de Compostela, Spain; 10 Instituto de Investigación Sanitaria de Santiago (IDIS), 15706 Santiago de Compostela, Spain; 11 Centro Singular de Investigación en Medicina Molecular y Enfermedades Crónicas (CIMUS), Universidade de Santiago de Compostela, 15782 Santiago de Compostela, Spain; 12 Facultad de Ciencias de la Salud, Universidad Fernando Pessoa Canarias, 35450 Las Palmas de Gran Canaria, Spain

**Keywords:** Human genetics, Genomics, Evolutionary biology

## Abstract

The Canary Islanders exhibit a unique genetic admixture, comprising European (EUR), North African (NAF), and sub-Saharan African (SSA) ancestries. We comprehensively characterized the full spectrum of small genetic variation in this population based on whole-exome sequencing data to further develop CIRdb. Our results revealed 387,555 variants, of which 15.1% were previously unreported, and we prioritized novel putative pathogenic variants exhibiting enrichment in respiratory, cardiovascular, and metabolic disorders. Genetic differentiation patterns provided fine-grained insights into within-archipelago differentiation. We revealed an EUR genetic ancestry enrichment around the 17q21.31 inversion (a pleiotropic locus associated with pulmonary, infectious, and immunological diseases) and a candidate selective sweep shared by Canary Islanders and the NAF population around prune exopolyphosphatase 1 (*PRUNE1*) gene, which is associated with body mass index, cardiovascular health, and metabolic and respiratory traits. Taken together, our findings show that CIRdb presents a valuable resource of exome-wide genetic variation in a population at the edge of Southwestern European genetic diversity.

## Highlights

• CIRdb is the first exome-wide catalog of genetic variation in the Canary Islands
• ROH patterns suggest distinct isolation histories in La Gomera and El Hierro
• A candidate selective sweep was identified in PRUNE1 in Canary Islanders

## Graphical abstract

**Figure:**
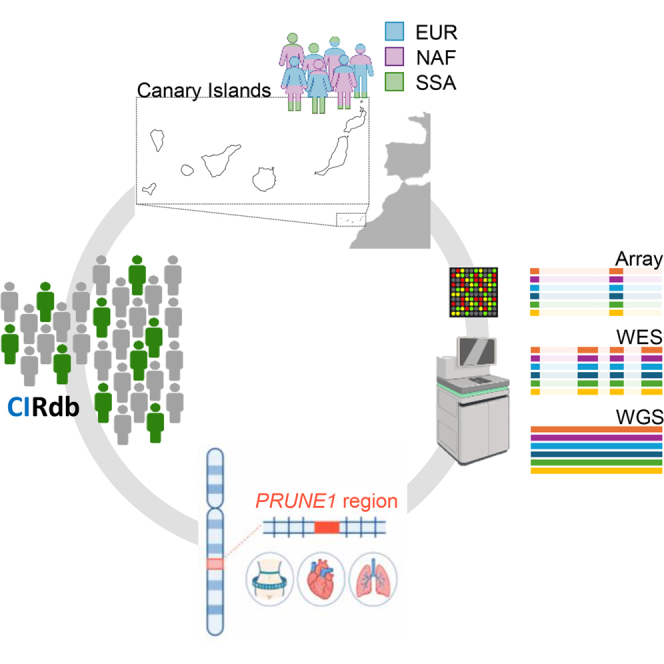

## Introduction

Democratization of next-generation sequencing has opened the possibility for international efforts aimed at deeply characterizing genetic variation in different human populations to improve biomedical research and clinical practice.^1^^,^^2^ The 1000 Genomes Project (1KGP) was among the first efforts to foster the discovery of genetic variation in diverse populations, and it revealed that our genome differs in roughly 5–6 million positions from the human reference genome.^3^ Many studies have evidenced the biomedical consequences of ignoring the genetic background of populations.^4^^,^^5^^,^^6^ These and others have served to establish the grounds to increase the diversity in catalogs of genetic variation to optimally represent population-specific particularities and to support personalized medicine strategies across populations worldwide.^7^ Many countries have developed their own genetic variation catalogs or conducted extensive genetic studies in control individuals^8^^,^^9^^,^^10^^,^^11^^,^^12^^,^^13^^,^^14^^,^^15^ by combining variant information and population features.^16^^,^^17^^,^^18^ In Spain, a pioneer study of 267 healthy individuals from Galicia and Andalusia highlighted the need to establish local population catalogs specifically in this country for an optimal understanding of genetic variation with clinical relevance.^18^

**Figure 1:**
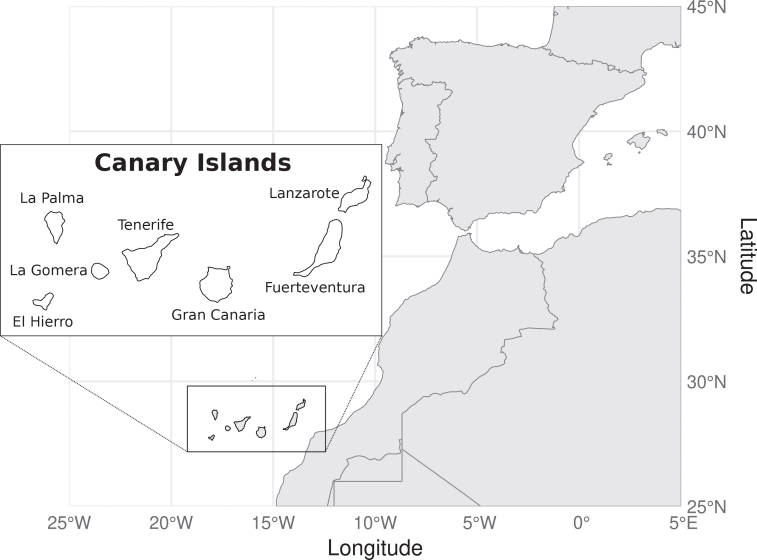
Geographical location of the Canary Islands

The Canary Islands archipelago (Spain) is located ∼100 km from the nearest point to the northwest African coast, at the southwestern edge of Europe (Figure 1). Originally, the Canary Islands were inhabited by aborigines whose most likely ancestral origin was in the Berber population from North Africa.^19^^,^^20^^,^^21^ Notwithstanding, during its conquest by Europeans in the 15th century, the archipelago was subjected to important events of admixture and aboriginals were displaced by European populations and imported slaves.^22^^,^^23^^,^^24^ As a result, the current Canary Islanders are a genetically admixed population of three main ancestries: European (EUR), North African (NAF), and sub-Saharan African (SSA). Mitogenomic studies in the current inhabitants have further unraveled fine-grained influences, such as those from Portuguese and Galicians.^25^ Furthermore, the geographic isolation of this population, the effects of sexual asymmetry (evidenced by the historically progressive decrease in male indigenous lineages),^26^^,^^27^ and multiple local adaptation events have finally modeled a particular genetic makeup where, on average, 75%–83% of the population have an EUR ancestry component, 17%–23% have an NAF ancestry component, and 2% or less have an SSA ancestry component.^28^^,^^29^ Individually, larger African ancestries were more recently recognized, with up to 38.2% NAF and 9.5% SSA components.^30^ However, although the Canary Island populations (aboriginal, historical, and modern) have been subjected to several analyses during the last two decades, studies have generally focused on a few genetic markers or small sample sizes.

On the basis of this and the particular genetic makeup of the current Canary Island populations, we developed the Canary Islanders’ reference database, termed CIRdb,^30^ as a detailed sequence-based exome reference catalog of genetic variation to allow disentangling population specificities with biomedical impact. In previous studies of the CIRdb cohort, we have exposed the overall design and methods to build the catalog, including population analyses based on SNP arrays and mitogenomes.^25^^,^^30^ Here, we analyze whole-exome sequencing (WES) data of the cohort to improve the population-specific reference catalog, also involving SNP array data and whole-genome sequencing (WGS) data for specific purposes. This enabled us to lay the foundation of resources for Genomic Medicine programs in the region and more in-depth analysis of the admixture and putative signals of adaptation.

## Results

### Exonic genetic variation in Canary Islanders

A total of 920 individuals and 387,555 WES variants were finally included in the CIRdb. The variants were annotated for several genetic elements, frequency across populations, and pathogenic potential (Table S1). No statistical differences were found in the average number of variants identified per individual across islands (one-way ANOVA, *p* = 0.558) (Table S2). Variants were classified into 357,846 single nucleotide variants (SNVs), 18,574 deletions, and 11,135 insertions, with an average transition/transversion (Ti/Tv) ratio in autosomes of 2.90 ± 0.04 (SD). The distribution by alternative allele frequency (AAF) strata was as follows: 47,493 common, 51,672 low-frequency, and 288,390 rare variants. There were 149,851 singletons and 56,188 doubletons in the rare AAF stratum. Thus, 74.4% of the variation had an AAF < 0.005, and nearly 40% of them were private to individuals. There were statistically significant differences between islands in the low-frequency and rare AAF strata (Figure S1) (one-way ANOVA, *p* < 2 × 10^−16^ for both comparisons; Duncan’s new multiple range tests, *p* < 0.05).

Heterozygosity analyses revealed that El Hierro had the highest number of homozygous variants among all the islands (one-way ANOVA, *p* = 1.95 × 10^−3^). Out of the 120,273 variants found in El Hierro, 43,613 were homozygous for the alternative allele in at least one individual, and 2,565 of those variants were found in homozygous state in at least 90% of individuals. We found statistically significant differences after Bonferroni correction in AAF for 16 variants between El Hierro and some of the other islands (Fisher’s exact test, *p* < 1.95 × 10^−5^) (Table S3). All these variants were cataloged as non-pathogenic by the InterVar tool, Combined Annotation Dependent Depletion (CADD), and the inferred impact in the Ensembl VEP annotation. Data from Open Targets Genetics linked several of the 16 variants to autoimmune diseases, cardiovascular traits, hair color, and skin browning processes, among others (Table S3). One of these variants is the missense variant rs2249265, which is functionally linked to *PTGFRN* and has been associated with type 2 diabetes (T2D),^31^ and it showed statistically significant differences in AAF between El Hierro and La Gomera.

**Figure 2:**
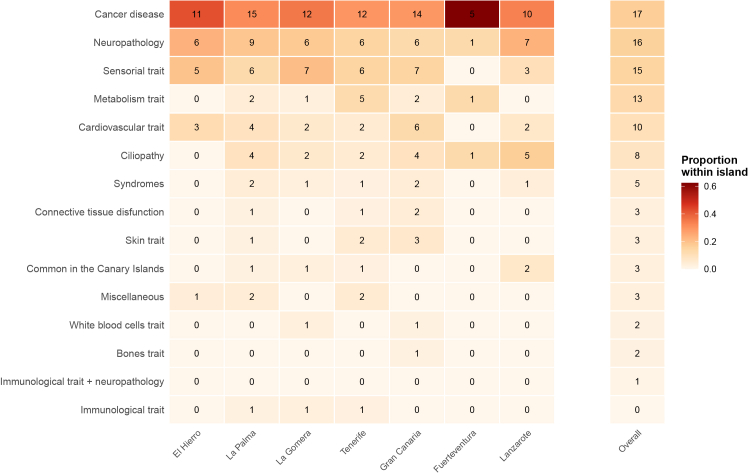
Enrichment analysis on the 2,068 putative pathogenic variants identified in the CIRdb catalog Annotations in Jensen Disease Ontology were evaluated. Box colors represent the within-island proportion of each category, while numbers indicate raw counts. Categories are ordered according to their overall frequency in the archipelago.

Considering pathogenicity classifications (combining the InterVar tool classification, C-score, and Mutation Significance Cutoff (MSC) values) and high impact together, 2,068 variants were identified globally as putatively pathogenic in CIRdb. These variants were enriched in the genes involved in oncological diseases as well as in neurodegenerative and intellectual disability based on Jensen Disease Ontology. A similar pattern was observed in a stratified analysis by island (Table S4). Overall, this observation evidenced enrichment in ciliopathies, neuropathologies, cardiovascular traits, and metabolic disorders. Sensory diseases, mainly those related to ocular conditions such as retinitis pigmentosa, cataract, and cone-rod or fundus dystrophy, were also significantly enriched (Figure 2).

**Figure 3:**
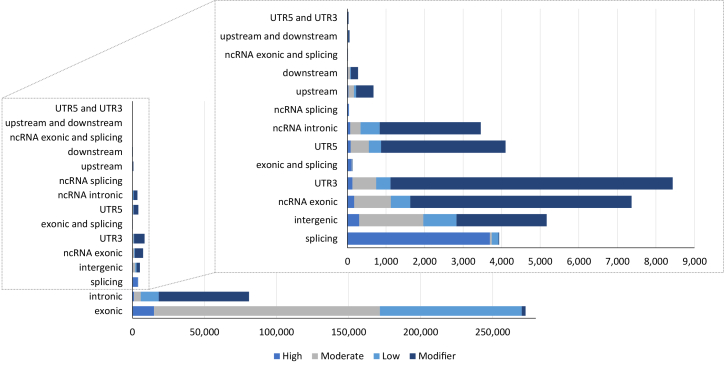
Distribution of whole-exome sequencing variants found in CIRdb based on genomic region and impact classification provided by Ensembl VEP Colors represent the following impact categories: dark blue for modifier impact, medium blue for high impact, gray for moderate impact, and light blue for low impact. ncRNA, noncoding RNA.

**Figure 4:**
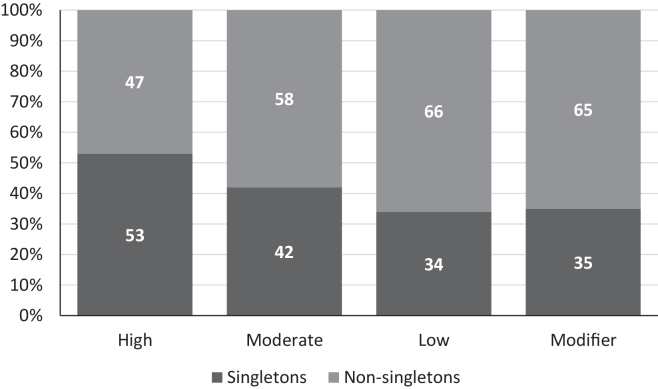
Proportion of singletons and non-singletons across different impact classifications found in CIRdb whole-exome sequencing data

When the affected genomic region and the impact classification provided by Ensembl VEP were simultaneously considered, 74.5% of variants in the exonic and intronic regions were within the four different impact categories (77.6% for high impact, 97.4% for moderate, 97.5% for low, and 74.9% for modifier) (Figure 3). Splicing with high impact was found for 3,695 variants (17.8% of the total). Moreover, in the splicing regions, nearly one out of every two variants (i.e., 49.1%) was a singleton. In line with the idea that variants of potentially higher functional importance are more likely to be singletons,^32^ we found a higher proportion of singletons among the high-impact variants (Figure 4). Further assessments of missense and loss-of-function variants (LoF) can be found in Figure S2 and Table S5.

### Novel variants in the CIRdb catalog

**Figure 5:**
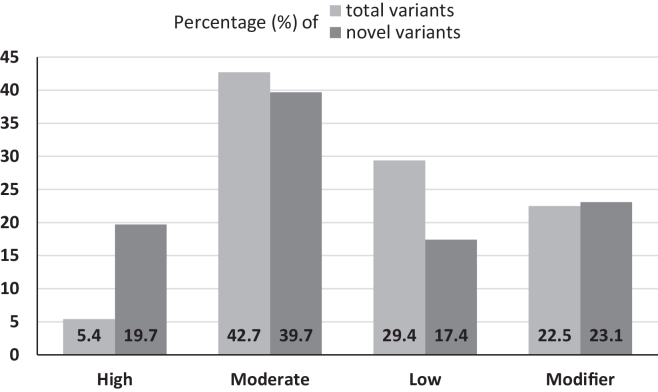
Distribution of total and novel variants based on impact classification found in CIRdb whole-exome sequencing data

A total of 15.1% of WES variants (*n* = 58,389) were categorized as novel because they were not present in dbSNP154, 1KGP, gnomAD exome v.2.1.1^1^ TOPMed v.5,^33^ or the HGDP-CEPH^34^ projects. La Palma island had the highest average of novel variants per individual, followed by Gran Canaria, Tenerife, La Gomera, Lanzarote, El Hierro, and Fuerteventura. However, statistically significant differences were observed only when compared with the last two islands (Duncan’s new multiple range test, *p* < 0.05). The novel variants were categorized by their AAF strata into 12 common variants, 1,311 with low frequency, 8,380 rare variants, 10,019 doubletons, and 38,667 singletons. Thus, 66% of the novel variants were also private to individuals. By impact, novel variants were enriched in the high-impact class when compared with all variants identified (Figure 5) and were distributed in 19.7% of high impact, 39.7% with moderate impact, 17.4% with low impact, and 23.1% classified as modifier. Additionally, 17.5% of the novel variants were also predicted to be LoF, and 1,242 (2.13%) of the novel variants were predicted as putatively pathogenic (Table S6). Overall, these variants were in genes associated with cardiovascular, immunological, respiratory, allergic, and sensory diseases, among others, and some of the affected genes were linked to skin pigmentation and sunburn. Furthermore, various novel putatively pathogenic variants affected genes that have been associated with COVID-19 susceptibility and severity, such as *TYK2* and *RAVER1*^35^ (Table S7).

### Shared genetic variation between Canary Islanders and North Africans

We found 10,758 WES variants exclusively shared between the Canary Islanders of CIRdb and the NAF WGS data, i.e., they were exclusive to both populations and were absent from the reference population dataset used in this study. We found no statistically significant differences in the number of these variants per island (one-way ANOVA, *p* = 0.456). However, La Palma showed the lowest number of the shared variants (622 ± [SD] 35) in comparison to all the other islands (one-way ANOVA, *p* = 0.002; Duncan’s new multiple range test, *p* < 0.05). Fuerteventura, Lanzarote, and El Hierro showed the largest number of these exclusive variants shared with NAF, followed by Tenerife and Gran Canaria. Their AAF classification was as follows: 1,178 common variants, 1,985 low-frequency variants, 3,442 rare variants, 1,506 doubletons, and 2,647 singletons. Most of them were classified as of moderate impact (41.3%), and none of these variants were predicted to be putatively pathogenic. Missense, synonymous, and intronic variants were the most abundant categories (Table S8). The 50 variants with high impact and the CADD > MSC similarly provided benign predictions or were variants of uncertain significance (VUS). Among these 50 variants, 13 showed statistically significant differences in AAF between the Canary Islands considered altogether and the NAF population after Bonferroni correction (Fisher’s exact test, *p* < 0.001) (Table S9). The AAF of five of these 13 variants was statistically different between some Canary Island populations and NAF. Interestingly, we did not find AAF differences in any of the 13 variants between Fuerteventura (one of the closest islands to the northwest African coast) and NAF. Nine out of these 13 variants were exonic to *GPRC6A*, *RP1L1*, *OR4X2*, *PKD1L2*, *KRT38*, *SIRPA*, and *DEFB126*. Interestingly, a genome-wide association study previously linked *DEFB126* variation with coronary artery disease in type 1 diabetes (T1D).^36^

### Exome-wide genetic differentiation of the Canary Island populations

**Figure 6:**
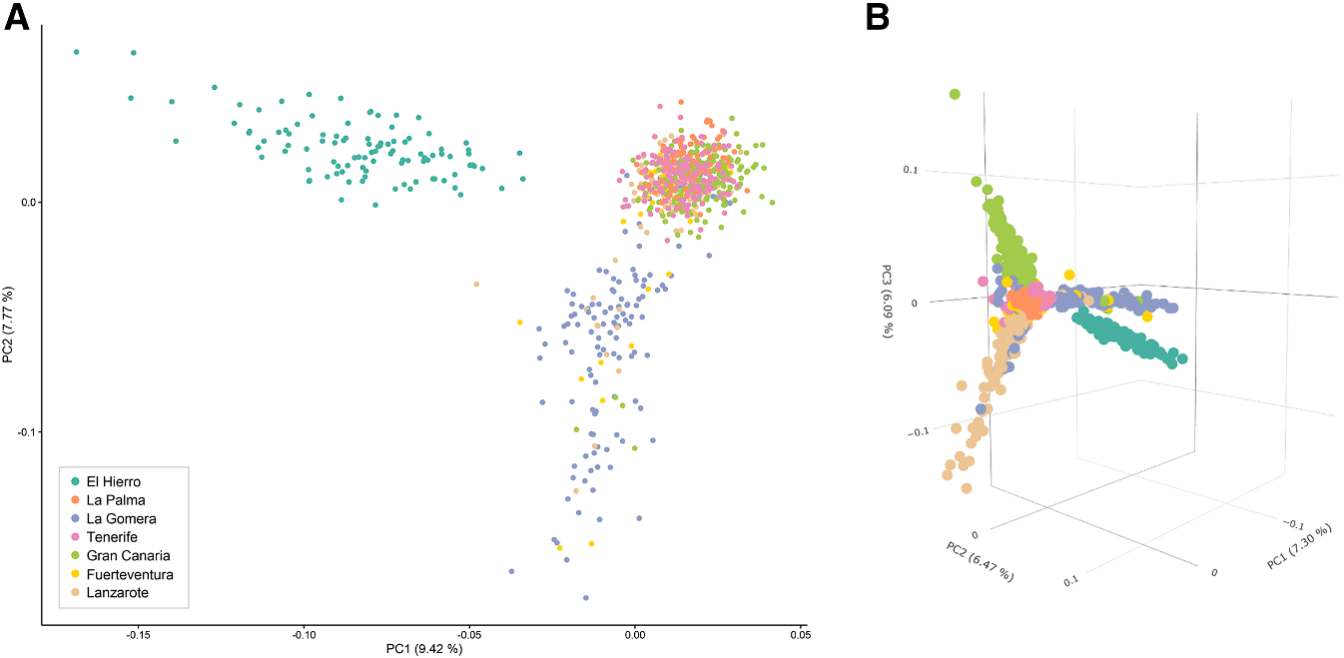
Representation of the exome-wide genetic variation in Canary Islanders (A) The first two principal components (PCs) of common variants (AAF > 0.05) explaining 17.2% of variability. (B) The first three PCs of rare variants (AAF < 0.005), excluding singletons and doubletons, explaining 19.9% of variability.

In the common frequency strata (AAF > 0.05), the first two principal components (PCs) clustered the Canary Islanders into three different groups: the first encompassed individuals from La Gomera, the second included individuals from El Hierro, and the third encompassed individuals from the other five islands (Mann-Whitney U test, *p* < 2.50 × 10^−16^ for all comparisons) (Figure 6A). In the rare AAF strata (AAF < 0.005), the third PC evidenced the differentiation of individuals from Gran Canaria from the rest of the islands (Mann-Whitney U test, *p* < 2.20 × 10^−16^ for the comparison of Gran Canaria against the rest of islands) (Figure 6B). When comparing against the reference populations, the Canary Islanders showed an intermediate position between the EUR and NAF populations (Mann-Whitney U test, *p* < 1 × 10^−22^ for all comparisons in the first PC), as observed elsewhere^30^^,^^37^ (Table S10). Tenerife and La Palma exhibited greater affinities with the EUR population, eminently with Iberian population in Spain (IBS), whereas La Gomera was the island clustering closer to NAF. Nevertheless, all pairwise comparisons among islands and EUR populations were significant (*p* < 1 × 10^−21^). EL Hierro, La Gomera, and NAF differentiated from the rest of populations for the first three PCs (Mann-Whitney U test, *p* < 6 × 10^−6^), except for the comparison of La Gomera and NAF in the second PC (Table S10).

**Figure 7:**
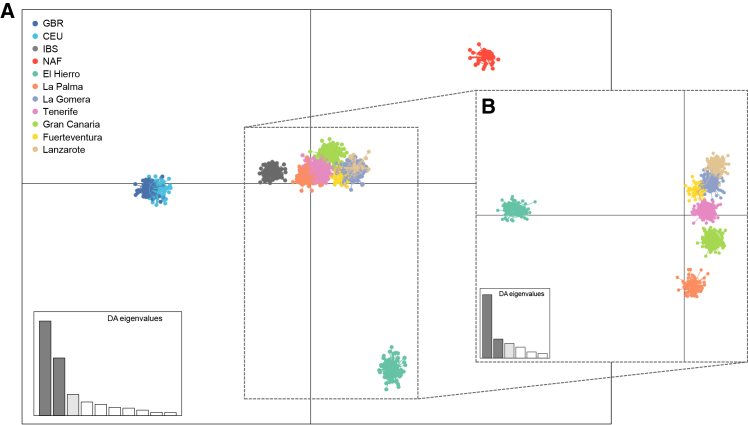
Discriminant analysis of principal components for all variants (A) Analysis including both Canary Islanders and reference populations (excluding outlier populations: SSA and FIN). (B) Analysis including only the Canary Islanders. LD1, horizontal axis; LD2, vertical axis. CEU, Utah Residents with Northern and Western European ancestry (1KGP); GBR, British (1KGP); IBS, Iberian population in Spain (1KGP); NAF, North African population.

To maximize discrimination among groups, we then relied on discriminant analysis of principal components (DAPC) with all WES variants, revealing El Hierro as a clear outlier when compared with the other Canary Islands (Figure 7). El Hierro showed the largest number of variants with statistically significant differences in AAF among comparisons within Canary Islands in the linear discriminant 1 (LD1; Fisher’s exact test after Bonferroni correction, *p* < 2.86 × 10^−5^), except in the rare AAF range. We identified 15 variants with statistically different AAF between El Hierro and five of the other islands. These 15 variants were in *FLT4*, *MALRD1*, C1orf159, *PPME1*, C12orf42, LOC101929058, *TMTC1*, *ABHD17A*, *SLC6A3*, *CEP85L*, *MRPL20-AS1*, *ZBTB17*, *RNF220*, *CXCR4*, and *GRIP2*. Intriguingly, some of these genes have been associated with cardiovascular traits: *FLT4* associates with coronary heart disease,^38^ *ZBTB17* with dilated cardiomyopathy,^39^ *MALRD1* with coronary artery disease in T1D,^40^ and *RNF220* with heart rate.^41^^,^^42^ Additionally, *MALRD1*, *TMTC1*, *CEP85L*, and *RNF220* have been involved in diverse respiratory conditions.^43^^,^^44^^,^^45^^,^^46^ In the rare AAF stratum, individuals from Gran Canaria clustered out when compared with the other populations (Figure S3A). Within the Canary Islands, El Hierro and Gran Canaria exhibited two separate clusters (Figure S3B).

### Genetic ancestry and ROH in CIRdb

**Figure 8:**
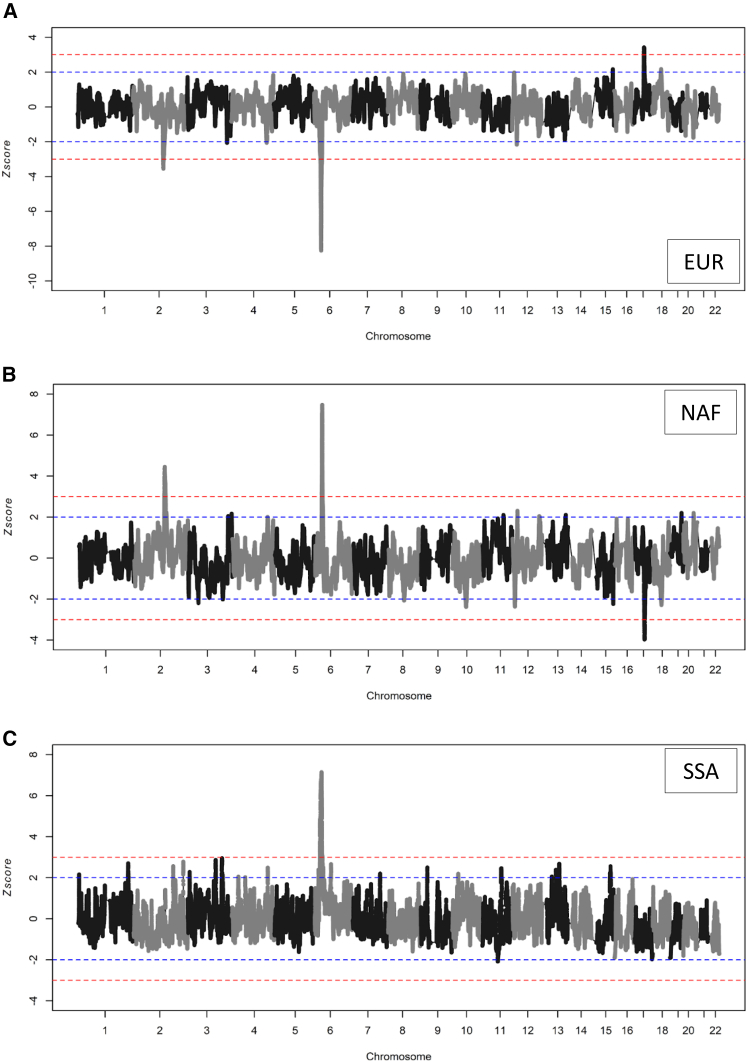
Genome-wide scan of deviations in local ancestry components in the current inhabitants of the Canary Islands from CIRdb (A) European. (B) North African. (C) Sub-Saharan African. Horizontal dashed lines (blue>|2|; red>|3|) indicate *Z* score thresholds.

Genetic ancestry proportions in CIRdb individuals were previously obtained based on the SNP array data.^30^ Further details of CIRdb individuals are provided in Table S11 and Figure S4, evidencing an overall picture of 77.6% ± 3.71% (SD) of a genetic ancestry associated with a component prevailing in EUR, 18.9% ± 3.10% (SD) of a component prevailing in NAF, and 3.52% ± 1.51% (SD) of a component prevailing in SSA. On the basis of local genetic ancestries, we previously identified genomic regions enriched in one of the ancestries and associated these regions with signals of selection.^37^ By leveraging a sample that nearly doubled that of a previous study, we revealed previously identified regions and a novel one showing a large deviation in the local genetic ancestry (Figure 8). The previously described regions included 2q21.3–2q22.1 (linked to lactase persistence) and 6p21.32 (in the HLA region), which were enriched in NAF and depleted in EUR ancestry, and 6p21.33, which was enriched in SSA ancestry. The novel region was located in 17q21.31 and was enriched in EUR and depleted in NAF ancestry (Table 1, supplemental information). This region overlaps with a well-known inversion site proposed to be under positive selection in EUR populations^47^ and with pleiotropic effects across diseases including severe respiratory diseases, among others.^48^^,^^49^^,^^50^^,^^51^

**Table 1:** Regions enriched or depleted in EUR, NAF, and SSA local ancestries in Canary Islanders

Ancestry	Regions	Lead SNP	*Z* score	pBH^a^	Mean local ancestry (%)	Genes
EUR	chr2: 135,338,640–137,893,387	rs6430600	−3.55	1.85 × 10^−4^	65.2	*DARS1*, *CXCR4*
	chr6: 28,881,660–35,468,873	(b)	−8.25	2.57 × 10^−4^	48.8	*TSBP1*
	chr17: 43,353,763– 44,977,142	rs1989480	3.42	1.41 × 10^−3^	89.6	*LINC02210-CRHR1*
NAF	chr2: 135,174,546–140,487,507	rs6430600	4.45	1.71 × 10^−4^	32.0	*DARS1*, *CXCR4*
	chr6: 30,074,163–33,956,165	(c)	7.47	2.35 × 10^−4^	40.8	*TSBP1*, *TSBP1-AS1*
	chr17: 43,068,547–45,308,047	rs1406072	−3.97	1.06 × 10^−3^	7.20	*KANSL1*
SSA	chr6: 23,934,383–35,337,197	rs3130985	7.14	9.80 × 10^−3^	12.4	*PSORS1C1*

a Benjamini-Hochberg-adjusted probability.

b rs17208363, rs9368712, rs9368714, rs9380289, rs9296021, rs3132958, rs10484560, rs9405090, rs3129949, rs3132959, rs742582, rs1003878, rs910052, rs1076712, rs17840113, rs9366793, rs9348882, rs9357140, rs16870056, and rs2022537.

c rs9268429, rs12528797, rs7758215, rs7757831, rs2894254, rs2395154, rs9268435, and rs28895023.

**Figure 9:**
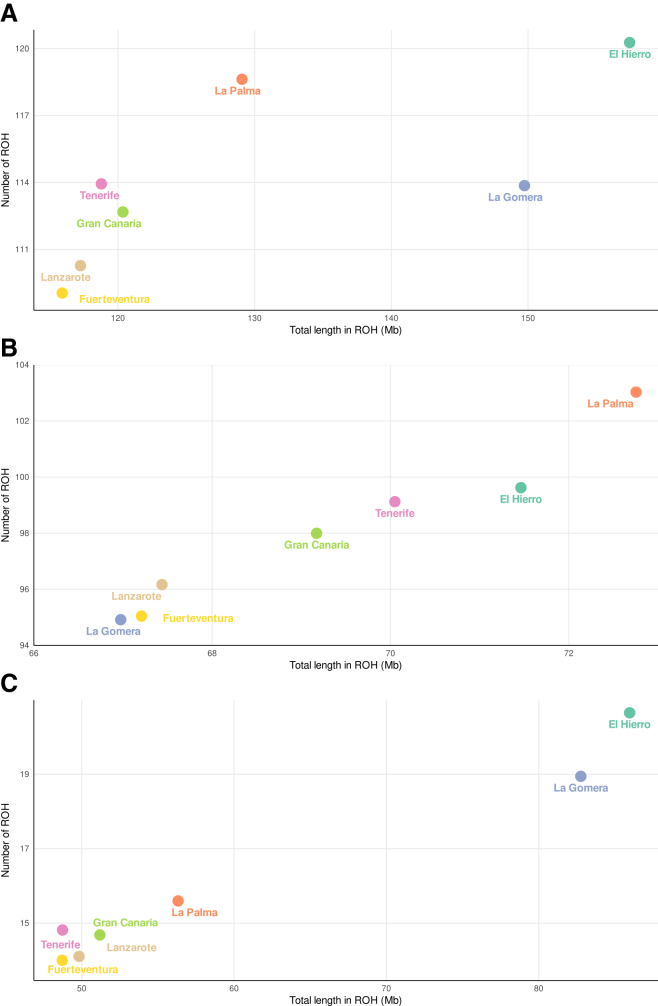
Average total ROH length (Mb) and the number of ROH fragments per island (A) All ROHs. (B) ROHs < 1.6 Mb. (C) ROHs ≥ 1.6 Mb.

We relied on the runs of homozygosity (ROH) estimates to assess isolation in the islands and were also represented in the reference population datasets for contextualization. In agreement with previous findings, El Hierro and La Gomera showed the highest estimates for the average total ROH, the total ROH length (Mann-Whitney U test, *p* < 1 × 10^−3^ for all pairwise comparisons), and the number of fragments in ROHs (Mann-Whitney U test, *p* < 1 × 10^−5^ for all pairwise comparisons) compared with all other islands (Figures 9, S5, and S6; Table S12). El Hierro and La Gomera showed a distribution of ROHs across length classifications ≥ 1.6 Mb comparable to Finnish, which are well-known isolates in Europe (Figures S5 and S6). Despite this, there were evident differences between El Hierro and La Gomera in their ROH distribution, with La Gomera showing relatively few short ROHs (<1.6 Mb), while El Hierro showed the largest number of long ROHs (≥1.6 Mb) (Figure 9).

### A scan for selective sweep around prune exopolyphosphatase 1 and a PheWAS of the exonic variant

**Figure 10:**
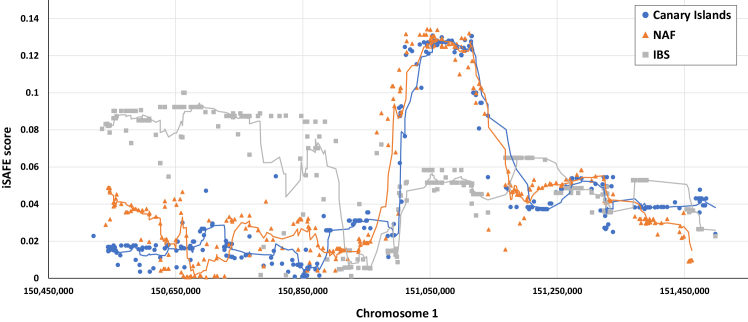
iSAFE scan of whole-genome sequencing data in the genomic region surrounding rs3738476 Lines represent the moving average for each population. IBS, Iberian population in Spain (1KGP); NAF, North African population.

**Figure 11:**
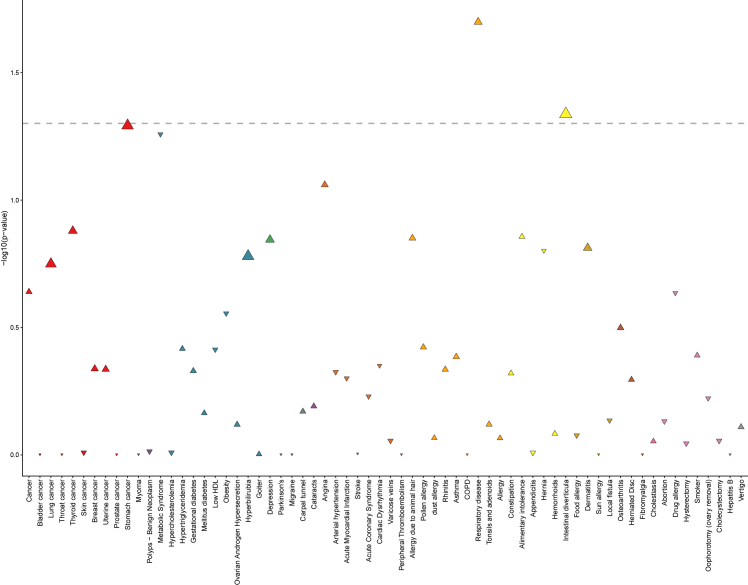
PheWAS of the rs3738476 variant Each triangle represents the *p* value of the association, pointing upward or downward depending on whether the variant is a risk or a protective factor. The size of the triangle corresponds to the odds ratio (OR), and its color indicates the respective ICD-9 category. The horizontal gray line represents the nominal significance threshold (*p* = 0.05).

Among the prioritized common variants in DAPC LD1, we identified 1,407 WES variants that showed no significant AAF differences between the Canary Islanders (altogether or for each island by separate) and the NAF population (Fisher’s exact test, Bonferroni-corrected *p* > 3.33 × 10^−5^). Six out of those variants showed statistically significant differences in AAF between the Canary Islanders and EUR populations (Fisher’s exact test, Bonferroni-corrected *p* < 3.33 × 10^−5^) despite being frequent variants (AAF > 10%) in the Canary Islands (Table 2; Table S13). Of those, we focused on rs3738476 in chromosome 1 (chr1), a variant exonic to prune exopolyphosphatase 1 (*PRUNE1*) gene that showed the largest difference in AAF between the Canary Island (87.2%) and NAF (91.2%) populations compared with the EUR population (AAF range: 49.5% and 52.7%), as quantified by the population branch statistics (PBS) genetic distance (0.303) and the pairwise F_ST_ distances between NAF or Canary Islanders vs. EUR population (Tables S14 and S15, respectively). To assess if variation in the region could be explained by a selective sweep, we conducted an integrated Selection of Allele Favored by Evolution (iSAFE) scan based on WGS data in a subset of the participants and in Iberian population in Spain (IBS) and NAF in the surrounding region of this variant (chr1:150,520,898–151,498,624) (Figure 10). The region with the highest iSAFE score was chr1:151,010,521–151,116,279 (Canary Islanders: iSAFE score > 0.12, peak at 0.131; NAF: iSAFE score > 0.11, peak at 0.136), while the signal tempered for IBS. This region harbors other 36 variants associated with body mass index (BMI)-related traits and functionally linked to nine genes involved in cardiovascular traits (*BNIPL*, C1orf56, *CDC42SE1*, *CTSS*, *LYSMD1*, *MLLT11*, *PRUNE1*, *SEM6C*, and *TNFAIP8L2*) and diabetes mellitus (*BNIPL*, C1orf56, *CDC42SE1*, *CTSS*, *MLLT11*, and *TNFAIP8L2*)^31^ (Table S16). We then conducted a phenome-wide association study (PheWAS) to examine possible associations between the rs3738476 variant at *PRUNE1* and the 66 available phecodes of participants (Figure 11). Although no associations with statistical significance were evidenced, nominally significant associations (*p* < 0.05) were found with respiratory disease, intestinal diverticula, and stomach cancer. Independent PheWAS results evidenced associations statistically dominated by diabetes (*p* = 6.8 × 10^−7^), fat (*p* = 9.58 × 10^−8^), anatomical adiposity distribution (*p* = 1.64 × 10^−7^), and BMI (*p* = 3.34 × 10^−14^). Besides these, association support was found only with respiratory traits, including forced vital capacity (*p* = 5.8 × 10^−25^) and asthma/atopy-related risks, including eosinophil counts (*p* = 7.8 × 10^−31^) and eczema/dermatitis (*p* = 1.5 × 10^−7^).

**Table 2:** Variants with similar alternative allele frequencies in Canary Islanders and the North African populations but differing from European populations

Chromosome	1	2	5	8	14	15
Position (hg19)	151,006,539	135,911,422	33,963,870	19,816,934	102,349,907	28,510,895
Reference allele	C	T	C	T	A	G
Alternative allele	A	C	T	C	G	A
Location	exonic	exonic	exonic	intronic	intronic	intronic
rsID	rs3738476^b^	rs17261772	rs26722^b^	rs301^b^	rs2720207	rs2240202
Gene	*PRUNE1*	*RAB3GAP1*^b^	*SLC45A2*	*LPL*	*PPP2R5C*^b^	*HERC2*^b^
Associated trait	body mass index	body fat percentage; LDL and total cholesterol levels; obesity	cutaneous melanoma, hair color, facial wrinkles (forehead)	diacylglycerol levels, metabolic syndrome	protein levels	cutaneous melanoma, hair, and eye color
AAF						
Canary Islands	0.872	0.557	0.114	0.237	0.313	0.142
CEU	0.495^a^	0.217^a^	0^a^	0.424	0.192	0.066
GBR	0.527^a^	0.275^a^	0.005	0.522^a^	0.104^a^	0.005^a^
IBS	0.519^a^	0.444	0.079	0.458^a^	0.201	0.187
NAF	0.913	0.558	0.173	0.163	0.317	0.192

AAF, alternative allele frequency; Alt, alternative allele; CEU, Utah Residents with Northern and Western European ancestry (1KGP); Chr, chromosome; GBR, British from England and Scotland (1KGP); IBS, Iberian population in Spain (1KGP); NAF, North African population; Ref, reference allele.

a Statistically significant differences with all Canary Island populations after Bonferroni correction (*p* < 3.33 × 10^−5^ for Fisher’s exact test).

b Variant or gene associated with the trait.

## Discussion

We developed CIRdb as the first exome-wide catalog of the natural genetic variation in the current inhabitants of the Canary Islands—the EUR population with the highest fraction of NAF ancestry reported to date. We showed that approximately 15.1% of the variants found were novel at the time the analyses were conducted, and this finding emphasized necessity for developing a population-specific catalog of genetic diversity in this archipelago.^30^ A similar scenario was observed in other populations like Cilento in South Italy^52^ and the Iranian population.^14^ These findings highlight the importance of genetic studies in diverse populations and the potential for unique discoveries. Strikingly, the island of La Palma exhibited the highest average of novel variants per individual, making it a compelling candidate for future studies on disease-causing variation. Our diverse analyses combining WES, SNP array, and WGS data and comparing the genetic background of the current Canary Islanders with other populations from Europe and Africa reinforced the recent admixed nature of the population and the footprints of population isolation, which were most salient in two of the smallest islands (El Hierro and La Gomera). In this context, the ROH may suggest an older or stratified history of isolation for the population of La Gomera compared with the prevalent signal of recent consanguinity and, possibly, of modern isolation for El Hierro population. This resource allowed us to identify genetic variation that could have significant biomedical implications for prevalent diseases in the Canary Islands, including metabolic, infectious, and respiratory diseases, among others, and in skin pigmentation and sunburn. We also identified a new region showing large deviations in local ancestry around a common inversion (17q21.31) known to be under positive selection in EUR populations and showing pleiotropic effects in human diseases. Our analyses, despite constrained by the lack of a formal framing under demographic scenarios models, also prioritized candidate selective sweep in the region around *PRUNE1* in Canary Islanders. This genomic region has been associated with BMI and metabolic and cardiovascular disorders, and we also provide evidence suggesting an association with respiratory traits. We expect that the CIRdb exome-wide catalog of the natural genetic variation in the Canary Islands will constitute a key resource to assist in genetic diagnosis of patients and in the identification of disease risks, as described elsewhere.^53^

The inhabitants of the Canary Islands have had a unique history of admixture and isolation in the southwestern EUR context. Although genetic isolation footprints have been well documented, at least for some of the islands such as El Hierro,^37^^,^^54^ its impact on the prevalence of genetic diseases in the population remains largely unexplored. Many studies support a higher prevalence of cardiovascular,^55^^,^^56^^,^^57^^,^^58^ respiratory, and allergic diseases^59^^,^^60^ in the Canary Islands compared with mainland regions of Spain. Some monogenic disorders have been linked to founder effects in particular islands, such as Fanconi anemia in La Palma,^61^ type 1 primary hyperoxaluria in La Gomera,^62^ and Wilson disease in Gran Canaria.^53^^,^^63^ This evidence is in line with our findings and the patterns of genetic differentiation in the exonic regions and the presence of private variants in current Canary Islanders.

There is support for the biomedical implications of the recent NAF genetic influences in the EUR population context.^29^ Accordingly, we prioritized common genetic variants, with AAF shared between Canary Islanders and NAF while diverged in EUR, including IBS, that were associated with the risk of cardiovascular disorders. Some of these were rs301 in *LPL* that is associated with insulin resistance and atherosclerosis^64^^,^^65^ and rs17261772 in *RAB3GAP1* that is associated with sudden cardiac arrest.^66^ We also prioritized genomic regions that showed putative evidence of a candidate selective sweep in the Canary Islanders and NAF but not in IBS, with a peak at a synonymous exonic variant (rs3738476) in *PRUNE1*. The risk allele rs3738476_A, which is near fixation in the Canary Islanders and NAF, was previously associated with BMI-related traits.^67^ The available evidence supports that the risk variant could affect gene function by creating a new “GGACU” sequence that would be susceptible to being methylated.^68^ However, caution is warranted in interpreting these findings given the lack of formal testing of the selective sweep under realistic demographic scenarios. A PheWAS of this variant in the Canary Islanders and a public, independent PheWAS also provided evidence of links with respiratory traits.

Taken together, we present CIRdb, the first catalog of genetic variation obtained by WES in current inhabitants of the Canary Islands. We found evident patterns of isolation in El Hierro and La Gomera. We also identified two genetic loci with well-known links with complex disease risk that exhibiting patterns suggestive of selective processes and shared genetic differentiation between NAF and Canary Islanders, distinct from those observed in EUR, providing a starting point for future functional and/or demographic modeling studies.

### Limitations of the study

The following constitute the main limitations of the study. First, WES was based on short-read technology applied to nearly 1,000 unrelated individuals reporting at least two ancestors born in the archipelago. This limited our ability to study ultra-rare variants and the optimal assessment of structural variation, which also pose significant risks for disease.^69^ Second, sequence variation at non-exonic regions remains largely uncovered, which necessitates whole-genome approaches to better understand disease architecture and provide further insights into population history.^70^ Third, because the study prioritized individuals without history of severe diseases as well as no cardiovascular, metabolic, immunologic, or cancer conditions, the PheWAS was based on a limited number of samples that were positively ascertained for the traits assessed. Despite this, independent PheWAS results provided support for some of the findings. Fourth, given that we used GRCh37/hg19 as the reference, our study may have missed information from important genomic regions that could be assessed by leveraging the most recent version of the human reference genome, i.e., T2T-CHM13.^71^ Fifth, because genetic sequence variation in North Africans is central to understanding the genetic diversity in the Canary Islanders, some of our results might have been affected by the limited diversity of NAF used as a reference due to their underrepresentation in global genetic studies.^72^^,^^73^ Sixth, as allele frequency differences were assessed by Fisher’s exact tests, these results should be interpreted with caution given that they did not account for linkage disequilibrium (LD) and local ancestry structure.

## Resource availability

### Lead contact

Requests for further information, resources, and reagents should be directed to and will be fulfilled by the lead contact, Carlos Flores (cflores.genomica@gmail.com).

### Materials availability

This study did not generate new unique reagents.

### Data and code availability

• SNP array and WES data are available from EGA (EGAS00001006050 and EGAS50000001726, respectively). Details for accessing the allele frequency data from the study can be found at https://github.com/genomicsITER/cirdb. Access to the raw genetic dataset is restricted to qualified researchers under institutional agreement to protect the privacy of the participants. Data access requests must be reviewed before release.
• Processing of WES data was carried out using an in-house pipeline based on GATK described elsewhere (https://github.com/genomicsITER/benchmarking/tree/master/WES).
• Any additional information required to reanalyze the data reported in this paper is available from the lead contact upon request.

## Acknowledgments

We would like to thank the support from our colleagues from the Teide-HPC Supercomputing facility (http://teidehpc.iter.es/en), which was funded by INP-2011-0063-PCT-430000-ACT (INNPLANTA program) from the 10.13039/501100003329Spanish Ministry of Economy and Competitiveness. This research was funded by 10.13039/501100004837Ministerio de Ciencia e Innovación (RTC-2017-6471-1; AEI/FEDER, UE) and the 10.13039/501100004587Instituto de Salud Carlos III (CB06/06/1088 and PI23/00980), which were co-financed by the European Regional Development Funds ‘A way of making Europe’ from the European Union; Fundación CajaCanarias and Fundación Bancaria “La Caixa” (2018PATRI20); Cabildo Insular de Tenerife (CGIEU0000219140); and the agreements OA17/008 and OA23/043 with Instituto Tecnológico y de Energías Renovables (ITER) to strengthen scientific and technological education, training, research, development, and scientific innovation in genomics, epidemiological surveillance based on massive sequencing, personalized medicine, and biotechnology. A.D.-d.U. was supported by a fellowship from the 10.13039/501100020636Spanish Ministry of Education and Vocational Training (grant number FPU16/01435). J.M.L.-S. was supported by Cabildo Insular de Tenerife and Consejería de Educación, Gobierno de Canarias (A0000014697).

## Author contributions

Conceptualization, C.F.; methodology, A.D.-d.U., R.C.-G., J.M.L.-S., and C.F.; investigation, A.D.-d.U., B.G.-G., I.M.-R., A. Corrales, R.G.-M., and A. Carracedo.; formal analysis, A.D.-d.U., L.A.R.-R., A.M.-B, J.M.L.-S., D.J., I.M.-R., and R.C.-G.; data curation, A.D.-d.U., L.A.R.-R., A.M.-B, R.G.-M., and D.J.; resources, M.d.C.R.-P., A.C.-d.-L., and A. Carracedo; supervision, C.F.; funding acquisition, C.F.; writing – original draft, A.D.-d.U. and C.F.; writing – review & editing, all authors.

## Declaration of interests

The authors declare that they have no potential conflicts of interest with respect to the authorship and/or publication of this article. The funders had no role in the design of the study; collection, analyses, or interpretation of data; writing of the manuscript; or in the decision to publish the results.

## Declaration of generative AI and AI-assisted technologies in the writing process

During the preparation of this work, the author(s) used DeepL Write in order to revise English grammar of the initial draft. After using it, the authors reviewed and edited the content as needed and take full responsibility for the content of the publication.

## STAR★Methods

### Key resources table

**Table:** 

REAGENT or RESOURCE	SOURCE	IDENTIFIER
**Biological samples**		

DNA samples	Cabrera de León et al.^74^	CDC of the Canary Islands

**Critical commercial assays**		

Illumina DNA Prep with Enrichment	Illumina, Inc.	20025524
Nextera DNA Exome	Illumina, Inc.	20020617

**Deposited data**		

Human reference genome NCBI build 37, GRCh37	Genome Reference Consortium	https://www.ncbi.nlm.nih.gov/assembly/GCF_000001405.13/
Human ancestral FASTA sequence, GRCh37	Ensembl	http://ftp.ensembl.org/pub/release-75/fasta/ancestral_alleles/
Reference whole-genome sequence data	1000 Genomes Project Consortium, Phase 3	https://www.internationalgenome.org/data-portal/data-collection
North African whole-genome sequence data	Serradell et al.^73^	https://www.ebi.ac.uk/ena/browser/view/PRJEB29142; https://ega-archive.org/studies/EGAS50000000299
Axiom Genome-wide Human CEU 1 Array data, CIRdb	Díaz-de Usera et al.^30^	https://ega-archive.org/studies/EGAS00001006050
Whole-exome sequencing data, CIRdb	This study	https://ega-archive.org/studies/EGAS50000001726

**Software and algorithms**		

GATK v.4	O’Connor and van der Auwera^75^	https://github.com/broadinstitute/gatk
ANNOVAR v18.04.16	Wang et al.^76^	https://annovar.openbioinformatics.org/en/latest/
GREAT	McLean et al.^77^	http://great.stanford.edu/public/html/splash.php
Enrichr	Kuleshov et al.^78^	https://maayanlab.cloud/Enrichr/
GeneSCF	Subhash and Kanduri^79^	https://github.com/genescf
Open Targets	Ghoussaini et al.^80^; Ochoa et al.^81^	https://www.opentargets.org/
PLINK v1.9	Chang et al.^82^	https://www.cog-genomics.org/plink/1.9/
AutoMap v1.0	Quinodoz et al.^83^	https://github.com/mquinodo/AutoMap
iSAFE v1.1.1	Akbari et al.^84^	https://github.com/alek0991/iSAFE

### Experimental model and study participant details

#### Sample selection, genotyping, and reference population datasets

The study was approved by the Research Ethics Committee of the Hospital Universitario Nuestra Señora de Candelaria (CHUNSC_2020_95) and performed according to The Code of Ethics of the World Medical Association (Declaration of Helsinki).

The CIRdb cohort was obtained from a total of 1,024 human participants (483 males, 541 females) that were selected from the largest general population cohort of the Canary Islands, the ‘CDC of the Canary Islands’ (Cardiovascular, Diabetes, and Cancer).^74^ Individuals who self-reported the absence of cardiovascular, metabolic, immunologic, or cancer diseases and had at least two generations of ancestors born on the same island were included in the present study. The island assigned to each individual corresponded to the island of origin of the four grandparents. However, owing to difficulties in sample collection, as was the case for Fuerteventura, this criterion was relaxed, allowing one of the four grandparents to be from a different island in the archipelago. Individuals whose grandparents were not from the same island were classified as No Island Assigned (NIA). These were considered for establishing the catalog, but not for the population, ancestry, or isolation analyses conducted in the study. Procedures for DNA extraction, SNP array genotyping, and quality control (QCs) were described previously.^30^^,^^37^ After QCs, 920 samples (439 males, 481 females) with paired WES and SNP array data were kept for further analyses: 106 from El Hierro, 99 from La Palma, 145 from La Gomera, 165 from Tenerife, 215 from Gran Canaria, 47 from Fuerteventura, 114 from Lanzarote, and 29 considered NIA.

Reference WGS data from 1KGP Phase 3^3^ were included in specific analyses to contextualize the genetic composition of the Canary Islanders in the EUR population landscape, which encompassed data from Finnish (FIN) (*N* = 99), British (GBR) (*N* = 91), and Utah Residents with Northern and Western European ancestry (CEU) (*N* = 99). In addition, data from the IBS (*N* = 107) was also considered for its genetic proximity to the Canary Islands population. For the African component, the SSA population was selected using the Yoruba population in Ibadan (Nigeria) (YRI) (*N* = 108) in the 1KGP as a proxy. The NAF (*N* = 32) was represented by data from individuals recruited at different locations north of the African continent.^73^ See the supplemental information for further details.

A total of 325,756 variants obtained by WES and 507,607 SNP array variants, a subset of all variants passing the QCs and included in the catalog, were used for the analyses that needed intersection with the population reference datasets. The SNP array data were only used for the ancestry and the ROH analyses.

### Method details

#### Whole-exome sequencing

Nextera DNA Exome Library Prep with a 350 bp insert size and Illumina DNA Prep with Enrichment kits were used for WES library preparation,^25^^,^^30^ according to the manufacturer’s instructions (Illumina Inc., San Diego, CA, USA). Pools of up to 12 dual-indexed libraries at 2 nM loading concentration were sequenced on Illumina HiSeq 4000 and NovaSeq 6000 Sequencing Systems (Illumina Inc.) with 75 bp and 100 bp paired-end reads, respectively, following the vendor’s guidance. All experiments used 1% PhiX Control V3 (Illumina Inc.) following the manufacturer’s recommendations.

Genomic data were processed at the TeideHPC Supercomputing facility (https://teidehpc.iter.es/en/home/), following an in-house bioinformatic pipeline described elsewhere,^85^ composed mainly of preprocessing, variant discovery, and QC steps. Additionally, GATK Best Practices^75^ for large cohorts were implemented in the joint variant calling stage using GATK HaplotypeCaller and the Genomic Variant Call Format (GVCF) mode. After variant calling, different filters were applied to refine the genetic variants included in the catalog (see supplemental information).

#### Variant annotation

Variant annotation was performed with ANNOVAR v18.04.16^76^ and The Ensembl Variant Effect Predictor (VEP) v100^86^ using GRCh37/hg19 as the reference genome.^87^ The software, databases, and plugins used for variant annotation are listed in Table S1.

Pathogenicity was defined according to the InterVar tool and the C-score reported by Combined Annotation Dependent Depletion (CADD).^88^ The C-score and the mutation significance cutoff (MSC) were used in combination so that the variant was cataloged as putative pathogenic when the C-score was higher than MSC (at 99% confidence). Statistical differences among the number of variants in the different analyses were assessed using the R environment,^89^ based on one-way ANOVA and Duncan’s new multiple range tests (*p* < 0.05). In particular cases, a Fisher’s exact test with subsequent Bonferroni correction was also used for variant comparisons. Enrichment analyses were performed using the Genomic Regions Enrichment of Annotations Tool (GREAT),^77^ Enrichr,^78^ and GeneSCF.^79^ The annotation of variants and genes with traits and diseases was performed using the GWAS Catalog,^90^ Open Targets Genetics,^80^ Open Targets Platform,^81^ GeneCards,^91^ and OMIM r20210128.^92^

#### Principal component analysis and discriminant analysis of principal components

To adapt to the WES data context, some modifications (i.e., variant exclusions due to call rate <0.80) from the aforementioned QC pipeline were done using PLINK v1.9.^82^ Principal Component Analysis (PCA) was performed using PLINK v1.9, after excluding variants located in regions of long-range LD and with high LD (window size = 50, step size = 5, r2 = 0.05), as previously described.^37^ For stratified analyses of genetic differentiation based on AAF range, variants were further sorted by allele frequency in common (AAF > 0.05), low frequency (0.005 ≤ AAF ≤ 0.05), and rare (AAF < 0.005). The same parameters (i.e., sample, variants, and AAF filters) were used for DAPC.^93^ Since rare variants consisting of singletons and doubletons behaved as outliers, they were removed from the PCA and DAPC assessments. A nonparametric Mann-Whitney U test was used to assess the differences between the first three PCs adjusting for the number of comparisons.

#### Genetic ancestry deviations and runs of homozygosity

Ancestry inferences based on ELAI v1.01^94^ were previously obtained for the CIRdb individuals using SNP array data^30^ given that it outperformed other commonly used local ancestry estimators in Canary Islanders.^95^ Ancestry analyses included WGS data from 423 individuals from the reference populations, which comprised all YRI, NAF, and EUR datasets except the IBS as detailed elsewhere.^30^^,^^37^ These estimates were based on a three-way admixture model of EUR, NAF, and SSA, assuming 14 generations since the last admixture event based on our previous observations.^37^ Extensive sensitivity analyses considering more diverse populations into SSA, switching the EUR reference by IBS, or balancing the number of individuals in all references (*n* = 32) provided similar ancestry estimates. Local ancestry block sizes and the average number of ancestry-related blocks in a haploid Canary Islander genome were calculated after removing centromeres and considering the flanking blocks as different elements to avoid overestimation of the block lengths. Deviations in the local parental genomic ancestries were individually assessed for each ancestry using the method developed by Zhu et al.^96^ Loci with a Z-score > |3| were prioritized by strongly deviating from the average^37^ and statistical significance with family-wise error rate (FWER)-corrected *p*-values assessed on those using the following procedure. Ancestry dosages across the genome were partitioned into 1 Mb windows (with 50% overlap of consecutive windows), and mean ancestry dosage per window was calculated. To preserve genome-wide ancestry proportions and autocorrelation while disrupting positional associations, the vector of window-level ancestry dosages was randomly permuted 50,000 times along the chromosome for each individual to generate a null distribution for each window. The observed ancestry dosage was then standardized using the local null mean and variance to obtain a locally calibrated Z-score. Empirical two-sided *p*-values were then computed by comparing the locally calibrated Z-score against the null distribution, and false discovery rate (FDR < 0.01) was controlled using the Benjamini–Hochberg procedure.

AutoMap^83^ v1.0-based ROH estimates under default settings, obtained according to previous procedures,^37^ were classified by the average length (1-2, 2-4, 4-8, 8-16, and ≥ 16 Mb). We also considered a simplified version of the classification proposed by Pemberton and colleagues (< 1.6 Mb and ≥ 1.6 Mb).^97^ In addition, the average total length of ROHs and the number of fragments in ROHs were studied per island using nonparametric Mann-Whitney U tests and Bonferroni correction adjustments (*p* < 2.38x10^−3^ to adjust for 21 comparisons).

#### Selective sweep analyses around the exonic variant of prune exopolyphosphatase 1 (*PRUNE1*) gene

As part of the DAPC, we first extracted variants contributing more to LD1 within the common frequency range. To evaluate the likely biomedical implications between ancestry and diseases, the AAF of these prioritized variants was analyzed using Fisher’s exact tests applying a Bonferroni correction to the comparisons between Canary Islanders, EUR, and NAF populations (*p* < 3.33x10^−5^ to adjust for 1,501 comparisons). For those variants with statistical differences, the number of individuals was first normalized to match the sample size of the smallest group (i.e., NAF). Subsequently, Weir and Cockerham’s F_ST_^98^ and Population Branch Statistics (PBS)^99^ estimates were obtained for each variant. Next, we used the software iSAFE v1.1.1^84^ to evaluate whether the variant with the highest F_ST_ estimation value in the comparisons with EUR subpopulations and its flanking regions showed patterns that could be reconciled with a selective sweep. An iSAFE scan was run in the region located one Mb upstream and downstream from the variant of interest, considering the IgnoreGaps flag and the default MaxFreq value (0.95) (see supplemental information). For this analysis, the *Homo sapiens* (GRCh37) ancestral FASTA sequence was obtained from Ensembl release 75 (http://ftp.ensembl.org/pub/release-75/fasta/ancestral_alleles/). Variants with the largest iSAFE scores were prioritized for functional annotation using the Variant to Gene (V2G) score^80^ (https://genetics.opentargets.org/).

#### Phenotype-wide association study of the PRUNE1 exonic variant

To perform a phenome-wide association study (PheWAS) analysis and given that previous studies support sufficient power for PheWAS of common variants with >200 cases,^100^ we examined associations between the previously identified statistically significant SNP in *PRUNE1* (rs3738476_C) and phenotypes based on the 9^th^ version of the International Classification of Diseases catalog (ICD-9 codes), using the PheWAS v0.99.6.1 R package. All the 920 individuals were mapped to 66 documented diseases in the ICD-9 codes that included at least 20 counts in the cohort and then converted into individual phenotype groups (‘phecodes’). Analyses included age and sex as covariates in all PheWAS models and statistical significance was set at *p* = 7.6x10^−4^ to correct for multiple comparisons. Public independent PheWAS results satisfying a *p* < 1.0x10^−5^ statistical significance were used to provide variant (GWASAtlas; https://atlas.ctglab.nl/PheWAS; accessed 29 April 2026) and gene level (ExPheWas; https://exphewas.statgen.org/; accessed 29 April 2026) support to CIRdb results.

### Quantification and statistical analysis

All statistical analyses and plot generation were performed using the R v4.0.2 environment.^89^

Statistical differences were assessed using one-way ANOVA and Duncan’s new multiple range tests, and Fisher’s exact test or Mann-Whitney U test with Bonferroni corrections when necessary. Since there is no specific test to assess the statistical significance of local genetic ancestry deviations in chromosomes, statistical significance in this case was obtained based on random permutations of window-level ancestry dosages controlling for the multiple comparisons with a Benjamini–Hochberg procedure. Statistical significance in PheWAS analysis was obtained with logistic regression models and results corrected for multiple comparisons based on the Bonferroni correction.

In the histograms, the bars represent means or percentages and the whiskers represent the standard deviation. The distribution of variants found based on genomic region and impact classification represents total counts in the cohort (Figure 3), while the global ancestry estimates are represented as pointwise estimates of each ancestry (Figure S4).

### Additional resources

More details and advances in the reference genetic catalog of the Canary Islands (CIRdb) and procedures for accessing data from the study can be found at https://github.com/genomicsITER/cirdb.

## Supplemental information

Document S1. Figures S1–S6, Tables S1, S2, and S8–S15, and supplemental methods

Table S3. Gene and trait annotations of the 16 variants with statistically significant differences in alternative allele frequencies within island populations Alt, alternative allele; Ref, reference allele. ^a^From GRCh37/hg19 coordinates; ^b^Most severe consequence; ^c^V2G score: Variant to Gene score; ^d^From Open Targets Genetics; ^e^From Open Targets Platform; ^f^El Hierro showed significant differences in the alternative allele frequency compared with these islands. El Hierro always showed higher alternative allele frequency values.

Table S4. Enrichment analysis by means of Enrichr

Table S5. GeneSCF annotation in the 123 genes, which encompassed variants above the mean (> two variants) considering HC LoF variants with C score > MSC and pLI >0.9

Table S6. Putative pathogenic novel variants

Table S7. GWAS catalog studies showing association with the genes linked to putative pathogenic variants

Table S16. Functionally related genes in the region around *PRUNE1* and their associations with diseases and traits ^a^From Open Targets Platform; ^b^From the GWAS Catalog; ^c^The highest *p* value was included.
