# Supplementary material for: Expanding CIRdb, a comprehensive catalog of whole-exome sequencing data of Canary Islanders
Source: iScience. 2026 Aug 10;29(8):117146. doi: 10.1016/j.isci.2026.117146 (PMC13486847; doi:10.1016/j.isci.2026.117146)
Supplement: Supplementary file 1 [file mmc1.pdf]

iScience, Volume *29*

Supplemental information

Expanding CIRdb, a comprehensive catalog of whole-exome sequencing data of Canary Islanders

Ana Díaz-de Usera, Luis A. Rubio-Rodríguez, Adrián Muñoz-Barrera, Jose M. Lorenzo-Salazar, Beatriz Guillen-Guio, David Jáspez, Almudena Corrales, Itahisa Marcelino-Rodríguez, María Del Cristo Rodríguez-Pérez, Antonio Cabrera-de León, Rafaela González-Montelongo, Raquel Cruz-Guerrero, Ángel Carracedo, and Carlos Flores

Supplementary information

Supplemental methods

Sample selection and SNP array genotyping

DNA was extracted from peripheral blood using the Blood genomicPrep Mini Spin Kit (Cytiva, Marlborough, MA, USA) following the manufacturer's recommendations. Prior to the sequencing process, genotyping was performed for the 587,352 variants contained in the Axiom® Genome-Wide Human CEU 1 Array (Affymetrix, Santa Clara, CA) supported by the National Genotyping Center (CeGen), Universidad de Santiago de Compostela Node. Image files were processed, and the genotyping quality controls (QCs) were conducted using the AffyPipe v2.10.0 open-source pipeline [S1] followed by additional standard QCs applied on samples using the R 3.2.2 environment and PLINK v1.9 as described elsewhere [S2]. Samples with sex discordances (as recorded in medical records versus genetically inferred), genotype call rate <0.95, outlier heterozygosity rate (average ± [4 x SD]), and kinship relatedness (PIHAT>0.2) were removed. We also filtered out non-autosomal variants, variants strongly deviating from Hardy-Weinberg equilibrium ($p<1x10^{-6}$), variants with a call rate <0.95, and variants with a minor allele frequency below 1% (MAF <0.01).

Reference population datasets

The North African population dataset (NAF) encompassed whole-genome data of 32 individuals from distinct countries gathered for a previous study sequenced with Illumina HiSeq 2000 using PE100 reads or HiSeq 4000 using PE150 reads [S3]. Before merging with EUR and SSA references, several filters were used on the NAF dataset: keeping sites with callable regions and read depth (DP) ≥10, flagged as PASS in the FILTER field, and Genotype Quality (GQ) ≥20. Additionally, multi-nucleotide variants (MNP) were converted to single-nucleotide variants (SNPs). Biallelic variants from the NAF dataset were merged with EUR and SSA populations using the *merge* command from BCFtools [S4], considering the identifier as CHR:POS:REF:ALT.

Whole-exome sequencing: library quantification

Libraries were checked using the Qubit™ dsDNA HS Assay Kit on a Qubit® 3.0 Fluorometer (Thermo Fisher Scientific, Waltham, MA, USA) and the Agilent D1000 and High Sensitivity D1000 ScreenTape Assays on a 4200 TapeStation system (Agilent Technologies, Santa Clara, CA, USA).

Whole-exome sequencing: processing of GVCF

Variant calling per sample was carried out with the GATK HaplotypeCaller and the output was provided in GVCF (Genomic Variant Call Format) mode. GVCF files were used as input for GATK GenotypeGVCFs obtaining one multisample genotyped raw VCF file. This raw VCF file was recalibrated with GATK Variant Quality Score Recalibration (VQSR) to reduce false positives and to reach a high level of sensitivity. The resulting recalibrated-multisample VCF from the VQSR step was split per-sample and used to refine the initial GVCF files, producing a refined-recalibrated final set which contained both refined-recalibrated-variant and non-variant sites in genomic VCF format (GVCF). Based on the splitted VCF data, non-variants sites in the GVCFs were refined per sample following next steps: a) if DP<10, a missing genotype (GT=./.) was assigned; b) if DP ≥10 and genotype did not include at least one

alternative allele (GT=./. or GT=0/0), genotype remained as detected by the method; c) if DP≥10 and genotype included at least one alternative allele, QUAL score normalized using Allele Depth (QD) was evaluated. Therefore, missing genotype (GT=./.) was assigned for QD<2 and homozygous reference (GT=0/0) was established as genotype for QD>2. These criteria were applied in sites corresponding to variants initially detected in the GVCF but not finally called in the joint-genotyped VCF.

Whole-exome sequencing: post-variant calling filters

The following filters were applied after variant calling:

1. Selection of sites located in the manifest file of target regions provided by the commercial target-capture solution;
2. Choice of callable regions;
3. Selection of sites with DP≥10;
4. Removal of mitochondrial chromosome; and
5. Filtering out of sites without a PASS flag and/or GQ<20.

Using BCFtools v1.10 [S4], individual GVCFs were merged into a multisample GVCF which was subjected to additional filters, including the conversion of multi-nucleotide variation into single-nucleotide variation and the selection of variant sites. Furthermore, variants susceptible to systematic sequencing bias in whole-genome analysis were removed [S5]. Variants with a low call rate (<0.80), significant deviation from Hardy-Weinberg equilibrium ($p<1x10^{-6}$), and with negative values for Inbreeding Coefficient (< -0.3), based on gnomAD v2.1 project [S6], were also excluded. Previously to the establishment of the final catalog of variants, a normalization step by means of BCFtools v1.10 was used to avoid discrepancies between variant callers and annotation software.

Whole-exome sequencing summary

Whole-exome sequencing of the CIRdb cohort provided an average of 8.41 Gbases ± 2.15 standard deviation (SD), 99.7 ± 0.40% (SD) of which were mapped against the reference genome and passed filters. The average on-target depth of coverage was 51.9 ± 19.6X (SD) (range: 15.7-163X). The average on-target breadth of coverage was 97.4 ± 1.09% (SD) at >1X, and 60.8 ± 13.8% (SD) at >30X.

Selective sweep analysis

Variants from whole-genome sequence data from 32 NAF, 66 Canary Islands, 58 IBS from 1KGP, and 10 YRI individuals randomly selected within 107 from 1KGP (acting as outgroup population) were assessed for the selective sweep analysis. For this purpose, the Canary Islanders whole-genome data was obtained with HiSeq 4000 using PE150 reads for a subset of the donors. Read alignment was carried out using the Burrows-Wheeler Aligner with Maximal Exact Matches algorithm (BWA-MEM) v0.7.12-r1039 [S7] and GRCh37/hg19 was used as the reference genome. The variant calling was performed using an in-house bioinformatics pipeline following the Genome Analysis Toolkit (GATK) v4 Best Practices guidelines [S8]. Biallelic SNPs flagged with PASS in the FILTER field were phased within the EagleImp-Web service [S9].

Supplemental results

Missense variants

A high proportion of variation was identified as missense variants (41.3%, moderate impact), meaning that there exists a prediction of an amino acid change in the final protein which may alter its function. There were no statistically significant differences among the number of missense variants among the islands (one-way ANOVA test, *p*=0.562). The second and the third most numerous variants, based on consequence classification, were synonymous and intron variants representing 25.5% and 14.1%, respectively. The rest of variants (19.2%) were distributed in 22 different groups. From a total of 159,940 missense variants, 21,001 were novel (i.e., not declared in dbSNP154, 1KGP, gnomAD exome, TOPMed v5, or HGDP-CEPH), with more than 98.4% classified as rare, including 14,520 singletons, and 3,589 doubletons. La Palma provided the highest average of missense novel variants per individual, being significantly different from El Hierro and Fuerteventura (one-way ANOVA test, *p*=0.045). Missense novel variants provided the following pathogenicity distribution based on InterVar annotation: 434 likely benign, 209 likely pathogenic, one likely pathogenic + VUS, one pathogenic variant, and 20,356 VUS. Applying the M-CAP score, 43.7% of VUS declared by InterVar could be cataloged such as possibly pathogenic variants based on threshold above 0.025.

Loss-of-function variants

Additionally, we performed an assessment of loss-of-function (LoF) variants. Out of the 387,555 total variants, 16,542 were labeled as high confidence (HC) by LOFTEE v1.0, of which 1,252 provided C-score values above MSC (99% CI) and pLI values above 0.9. No differences in the presence of LoF variants were observed among islands (ANOVA test, *p*=0.373). HC LoF variants with C-score value above MSC (99% CI) and pLI >0.9 were distributed on 776 genes, where a total of 123 genes encompassed variants above the mean (i.e., two variants per gene). According to the first 20 biological processes identified by GO analysis, enrichment in these 123 genes supported links to aorta development, regulation of catalytic and lipid kinase activity, and aortic valve and ventricular compact myocardium morphogenesis, among others (Figure S4, Table S5).

Prioritization analysis from the DAPC LD1

As observed for rs3738476 in *PRUNE1*, only a few variants for CEU (2), GBR (4), and IBS (1) populations, showed clear differences in AAF compared to the Canary Islands data considered in aggregate (**Table 2**). The exonic variant in *RAB3GAP1* gene (rs17261772) was related to traits which have a close relationship with cardiovascular and respiratory systems, such as body fat percentage, LDL and total cholesterol levels or obesity, and asthma. Additionally, this variant has been established as a protein quantitative trait locus (pQTL) and/or expression quantitative trait locus (eQTL) of some genes implicated in lactose metabolism (i.e., *LCT, MCM6,* and *DARS1* genes). Variants in chromosomes 5 (rs26722) and 15 (rs2240202) are involved in skin, hair, and eye pigmentation, with rs2240202 acting as an eQTL of *HERC2* and *OCA2,* both genes in line with the same traits. The intronic variant in *LPL* gene (rs301) plays a main role in lipoprotein metabolism as well as in some complications derived from it such as coronary artery or heart diseases.

Local ancestry blocks

The EUR-related region in chr2 displayed an average local ancestry of 65.2% for its lead SNP which was an intergenic variant located between the *DARS1* and *CXCR4* genes (2q21.3 and 2q22.1), whereas the regions in chr6 (with all lead SNPs located within *TSBP1*, 6p21.32, including two exonic variants and 18 intronic) and in chr17 (lead SNP located in an intronic region of the *LINC02210-CRHR1* gene, 17q21.31) exhibited an average local ancestry of 48.8% and 89.6%, respectively (**Table 2**). The loci with clear deviation in NAF ancestry covered regions from chr2 (2q21.3 and 2q22.1) (with the same lead SNP that from EUR-related peak, with an average local ancestry of 32.0%), chr6 (showing all lead SNPs as intergenic variants flanked by *TSBP1* and *TSBP1-AS1*, 6p21.32, with an average local ancestry of 40.8%), and chr17 (providing the lead SNP with an average local ancestry of 7.20% and within the *KANSL1* gene, 17q21.31). The locus enriched in SSA ancestry was located in *PSORS1C1* gene in chr6 (6p21.33), where the intronic lead SNP provided an average local ancestry of 12.4%.

The lead SNP from chr2 (rs6430600) has been related to *LCT* and *MCM6* genes by means of the Variant to Gene (V2G) score [S10], being involved in metabolism of the lactose. Lactase persistence, one of the strongest examples of positive selection [S11] on the human genome, is a heterogeneous trait whose worldwide distribution range from 5% to almost 100%. Despite the interest aroused by this trait, nowadays there are populations that still are underrepresented in this context (i.e., North African population). Thus, the signal observed in our study may pinpoint a positive selection in this region caused by North African genomic ancestry. Strikingly, two of the prioritized variants from DAPC, for both scenarios considered, were located in this NAF-enrichment region in chr2 (rs61731939 and rs17261772). When regions in chr6 were considered, different variants acted as eQTLs of five of the eight classical HLA alleles (*HLA-B*, *HLA-C*, *HLA-DBQ1*, *HLA-DRB1*, *HLA-DQA1*) and of several non-classical HLA alleles. The human leukocyte antigen genes are located in the major histocompatibility complex (MHC) which is the most variable region in the human genome, characterized by a high degree of polymorphism and numerous functional genes. Studies around these genomic coordinates have emphasized an important selective process, not only in a balancing scenario such as could be observed in European and African populations [S12, S13], but also in positive selection for African populations [S14] and in negative selection in a population from North America [S15]. Therefore, peaks in chr2 and chr6 signals may be explained as the result of putative selective processes in these regions within the Canary Islanders genome.

Supplemental tables

Table S1. Software, databases, and plugins used for variant annotation.

Software	Database / Plugin	Release / Version	Annotation information
ANNOVAR	RefGene	r20190929	Genes
	cytoBand	r20180813	Regions
	ClinVar	r20210501	Phenotypes
	dbnsfp	v4.1a	rsID number
	gnomAD exome	v2.1.1	Allele frequency in other populations
	1KGP	r2015aug	Allele frequency in diverse populations
The Ensembl VEP	dbSNP human	Build 154	rsID number
	Combined Annotation Dependent Depletion (CADD)	v1.6	Pathogenicity prediction score
	Loss-Of-Function Transcript Effect Estimator (LOFTEE)	v1.0	Pathogenicity prediction score
	Probability of a gene being loss-of-function intolerant (pLI)	r0.3	Pathogenicity prediction score
InterVar	-	-	Clinical interpretation of genetic variants based on the 2015 ACMG-AMP Guidelines
BCFtools	Mendelian Clinically Applicable Pathogenicity (M-CAP)	v1.4	Pathogenicity likelihood score
	Mutation Significance Cutoff (MSC)	v1.6	Gene-level and gene-specific phenotypic impact cutoff values
	Online Mendelian Inheritance in Man (OMIM)	r20210128	Relationships between human genes and phenotypes

Table S2. Total number of variants per island and average per island, per individual. SD, standard deviation.

Island	Samples	Variants per island	Average of variants per island per individual (± SD)
El Hierro	106	120,273	25,729 ± 815
La Palma	99	143,035	25,413 ± 1,143
La Gomera	145	163,321	25,601 ± 1,709
Tenerife	165	184,372	25,632 ± 678
Gran Canaria	215	208,919	25,714 ± 1,488
Fuerteventura	47	111,100	25,664 ± 842
Lanzarote	114	152,656	25,478 ± 2,257
NIA	29	94,131	-

Table S8. Distribution of consequence considering total, not shared, non-exclusively shared, and exclusively shared variants with North African population, within the reference population dataset used. Consequences were extracted from Ensembl VEP.

Consequence	Total	Not shared with NAF	Shared with NAF	Non-exclusively shared with NAF	Exclusively shared with NAF
Splice acceptor variant	4,771	4,603	168	131	37
Splice donor variant	1,428	1,227	201	170	31
Stop gained	4,313	3,806	507	418	89
Frameshift variants	9,434	8,735	699	472	227
Stop lost	351	249	102	89	13
Start lost	484	346	138	123	15
In frame insertion	1,774	1,557	217	130	87
In frame deletion	3,709	3,211	498	323	175
Missense variant	159,940	122,018	37,922	33,740	4,182
Protein altering variant	217	213	4	2	2
Splice region variant	14,766	10,966	3,800	3,441	359
Incomplete terminal codon variant	13	9	4	3	1
Stop retained variant	108	74	34	31	3
Synonymous variant	98,885	64,426	34,459	31,652	2,807
Coding sequence variant	24	18	6	5	1
Mature miRNA variant	84	52	32	28	4
5 prime UTR variant	4,610	3,166	1,444	1,324	120
3 prime UTR variant	9,188	6,242	2,946	2,648	298
Non coding transcript exon variant	17,351	11,545	5,806	5,151	655
Intron variant	54,488	37,776	16,712	15,124	1,588
Upstream gene variant	858	579	279	236	43
Downstream gene variant	410	263	147	133	14
TF binding site variant	5	4	1	1	0
Regulatory region variant	81	54	27	26	1
Intergenic variant	263	158	105	99	6

Table S9. Alternative allele frequencies (AAF) for common variants exclusively shared between Canary Islands and NAF populations, within the reference population dataset used, showing statistical differences in AAF both among islands and between the islands and the NAF population.

Chr	Position	Ref	Alt	rsID	EH	LP	LG	TF	GC	FV	LZ	NAF
6	117,113,762	T	TA	rs371464745	0.129	0.032	0.125	0.076	0.097	0.109	0.111	0.116
7	6,713,940	CAG	C	rs58381208	0.132	0.106	0.209	0.155	0.137	0.298	0.093	0.205
8	10,467,651	C	CCTCTCTTCTT	rs773894295	0.033*	0.035*	0.072*	0.070*	0.072*	0.064	0.071*	0.214
8	23,282,562	A	AGTATT	rs201841918	0.042	0.121	0.052	0.091	0.097	0.043	0.144	0.061
11	48,266,736	C	G	rs7120775	0.208	0.106	0.049	0.097	0.158	0.032	0.086	0.091
13	100,517,195	CTG	C	rs3831038	0.052	0.122	0.100	0.110	0.161	0.237	0.175	0.144
16	81,242,148	GTT	G	rs55980345	0.491*	0.379	0.321	0.355	0.381	0.426	0.355	0.259
17	39,595,484	G	A	rs148768443	0.074	0.054	0.036*	0.060	0.043*	0.057	0.073	0.154
20	126,155	GCAAA	G	rs11467497	0.325	0.242*	0.184*	0.226*	0.223*	0.234	0.232*	0.438
20	126,310	ACC	A	rs11467417	0.767	0.665	0.624	0.647	0.628	0.660	0.638	0.732
20	1,895,949	A	AGT	rs148409797	0.193	0.332	0.322	0.278	0.306	0.319	0.407	0.307
20	1,895,950	CCT	C	rs749337996	0.203	0.361	0.343	0.275	0.312	0.319	0.405	0.307
22	22,707,728	C	T	rs148013584	0.125*	0.066*	0.173	0.146*	0.111*	0.202	0.147*	0.320

Alt, alternative allele; Chr, chromosome; EH, El Hierro; FV, Fuerteventura; GC, Gran Canaria; LG, La Gomera; LP, La Palma; LZ, Lanzarote; NAF, North African population; Ref, reference allele; TF, Tenerife. * Significant differences in AAF after Bonferroni correction with NAF population (p <0.001 for Fisher's exact test).

Table S10. Nonparametric Mann-Whitney U-test results for the principal components (PC) 1, PC2, and PC3 in PCA for Canary Islanders and the reference populations, excluding FIN and SSA.

	CEU	GBR	IBS	EH	LP	LG	TF	GC	FV	LZ
**PC1**										
**GBR**	0.001	-	-	-	-	-	-	-	-	-
**IBS**	5.35×10^{-35}	9.70×10^{-34}	-	-	-	-	-	-	-	-
**EH**	8.65×10^{-35}	2.34×10^{-33}	1.10×10^{-33}	-	-	-	-	-	-	-
**LP**	8.44×10^{-33}	1.58×10^{-31}	2.31×10^{-24}	1.68×10^{-23}	-	-	-	-	-	-
**LG**	9.48×10^{-39}	5.75×10^{-37}	3.66×10^{-40}	1.98×10^{-33}	5.18×10^{-36}	-	-	-	-	-
**TF**	9.78×10^{-42}	1.15×10^{-39}	1.05×10^{-36}	9.91×10^{-12}	2.37×10^{-7}	2.12×10^{-43}	-	-	-	-
**GC**	1.58×10^{-45}	4.61×10^{-43}	1.15×10^{-44}	0.033	2.88×10^{-25}	8.12×10^{-29}	7.07×10^{-16}	-	-	-
**FV**	3.99×10^{-22}	1.47×10^{-21}	5.48×10^{-22}	1.63×10^{-10}	8.05×10^{-19}	1.02×10^{-7}	4.54×10^{-16}	3.04×10^{-4}	-	-
**LZ**	1.79×10^{-34}	4.56×10^{-33}	1.64×10^{-35}	2.21×10^{-28}	6.42×10^{-32}	4.54×10^{-8}	1.48×10^{-35}	6.92×10^{-16}	0.006	-
**NAF**	6.91×10^{-24}	3.18×10^{-23}	1.75×10^{-24}	2.88×10^{-24}	2.61×10^{-23}	7.67×10^{-26}	2.42×10^{-27}	8.14×10^{-29}	1.96×10^{-17}	4.39×10^{-24}
**PC2**										
**GBR**	0.005	-	-	-	-	-	-	-	-	-
**IBS**	4.49×10^{-35}	8.86×10^{-34}	-	-	-	-	-	-	-	-
**EH**	8.65×10^{-35}	2.34×10^{-33}	4.10×10^{-36}	-	-	-	-	-	-	-
**LP**	1.12×10^{-32}	1.58×10^{-31}	3.50×10^{-12}	1.78×10^{-33}	-	-	-	-	-	-
**LG**	9.48×10^{-39}	5.75×10^{-37}	1.04×10^{-37}	0.402	3.91×10^{-32}	-	-	-	-	-
**TF**	9.78×10^{-42}	1.15×10^{-39}	8.03×10^{-31}	9.69×10^{-43}	7.22×10^{-6}	2.31×10^{-39}	-	-	-	-
**GC**	4.72×10^{-45}	9.50×10^{-43}	1.48×10^{-12}	2.57×10^{-44}	0.206	1.77×10^{-45}	1.26×10^{-11}	-	-	-
**FV**	3.99×10^{-22}	1.47×10^{-21}	1.61×10^{-21}	6.62×10^{-12}	2.88×10^{-14}	2.27×10^{-8}	6.35×10^{-11}	1.87×10^{-17}	-	-
**LZ**	1.79×10^{-34}	4.56×10^{-33}	9.58×10^{-35}	9.32×10^{-25}	5.35×10^{-25}	3.28×10^{-17}	2.43×10^{-22}	4.62×10^{-34}	0.773	-
**NAF**	3.32×10^{-22}	1.36×10^{-21}	9.30×10^{-23}	6.50×10^{-6}	1.13×10^{-21}	0.001	2.35×10^{-25}	5.37×10^{-26}	3.05×10^{-10}	1.23×10^{-18}
**PC3**										
**GBR**	0.979	-	-	-	-	-	-	-	-	-
**IBS**	1.06×10^{-30}	4.29×10^{-30}	-	-	-	-	-	-	-	-
**EH**	1.58×10^{-33}	3.77×10^{-32}	8.38×10^{-35}	-	-	-	-	-	-	-
**LP**	5.18×10^{-32}	4.95×10^{-31}	1.65×10^{-8}	2.50×10^{-32}	-	-	-	-	-	-
**LG**	6.80×10^{-14}	2.60×10^{-13}	1.15×10^{-14}	1.17×10^{-17}	4.56×10^{-14}	-	-	-	-	-
**TF**	2.47×10^{-40}	1.63×10^{-38}	9.84×10^{-17}	2.05×10^{-41}	0.068	1.36×10^{-18}	-	-	-	-
**GC**	3.18×10^{-42}	6.05×10^{-40}	4.97×10^{-44}	3.31×10^{-45}	3.35×10^{-37}	1.66×10^{-35}	5.05×10^{-50}	-	-	-
**FV**	3.96×10^{-9}	6.46×10^{-9}	2.05×10^{-7}	3.78×10^{-16}	2.03×10^{-4}	1.77×10^{-6}	4.53×10^{-4}	4.46×10^{-11}	-	-
**LZ**	7.71×10^{-21}	5.17×10^{-20}	2.21×10^{-21}	3.23×10^{-32}	2.10×10^{-18}	2.14×10^{-20}	4.30×10^{-22}	0.207	1.14×10^{-6}	-
**NAF**	3.32×10^{-22}	1.36×10^{-21}	9.30×10^{-23}	1.48×10^{-22}	1.13×10^{-21}	4.63×10^{-24}	2.41×10^{-25}	2.12×10^{-25}	3.31×10^{-16}	1.30×10^{-21}

CEU, Utah Residents with Northern and Western European ancestry; EH, El Hierro; FV, Fuerteventura; GC, Gran Canaria; GBR, British; IBS, Iberian population in Spain; LG, La Gomera; LP, La Palma; LZ, Lanzarote; NAF, North African population; TF, Tenerife. Significant differences in PCs were established at $p < 9.09\times10^{-4}$ considering a Bonferroni correction.

Table S11. Genomic global ancestry proportions (%) obtained by ELAI in the Canary Islanders assuming 14 generations.

	EUR			NAF			SSA		
**Island**	**Min**	**Mean±SD**	**Max**	**Min**	**Mean±SD**	**Max**	**Min**	**Mean±SD**	**Max**
El Hierro	69.9	75.5±1.91	81.7	16.9	22.2±1.76	27.6	1.29	2.34±0.55	4.04
La Palma	77.0	81.8±1.99	86.9	12.2	16.0±1.61	19.9	0.90	2.26±0.81	4.72
La Gomera	66.1	73.7±2.07	80.7	16.9	21.1±1.89	26.2	2.35	5.15±1.36	10.8
Tenerife	74.5	80.6±2.15	85.2	12.3	16.9±1.83	21.2	0.77	2.48±0.77	5.59
Gran Canaria	70.5	79.4±2.39	84.6	13.2	16.5±1.64	24.4	1.37	4.12±1.48	8.73
Fuerteventura	69.9	74.6±2.48	79.8	17.4	21.8±1.91	25.9	1.95	3.62±0.97	6.05
Lanzarote	69.6	74.6±2.21	81.6	16.0	21.5±1.85	26.0	2.06	3.95±0.87	6.28
Total	66.1	77.6±3.71	86.9	12.2	18.9±3.10	27.6	0.77	3.52±1.51	10.8

EUR, European; NAF, North African; SSA, sub-Saharan African.

Table S12. Nonparametric Mann-Whitney U-test resulting p-values for the number of fragments in runs of homozygosity (ROHs) in the Canary Islands comparisons.

	El Hierro	La Palma	La Gomera	Tenerife	Gran Canaria	Fuerteventura
**La Palma**	**0.002**	-	-	-	-	-
**La Gomera**	**2.21×10^{-5}**	0.228	-	-	-	-
**Tenerife**	**5.89×10^{-7}**	0.113	0.834	-	-	-
**Gran Canaria**	**5.95×10^{-12}**	**2.15×10^{-4}**	0.009	0.010	-	-
**Fuerteventura**	**8.27×10^{-7}**	0.003	0.030	0.033	0.551	-
**Lanzarote**	**5.32×10^{-15}**	**1.37×10^{-7}**	**7.79×10^{-6}**	**4.11×10^{-6}**	0.019	0.363

Significant differences after Bonferroni correction are shown in bold (established at $p < 2.38 \times 10^{-3}$).

Table S13. Alternative allele frequency (AAF) per island for variants with AAF similar to the North African (NAF) population and different from the European (EUR) populations.

**Chr**	1	2	5	8	14	15
**Position**	151,006,539	135,911,422	33,963,870	19,816,934	102,349,907	28,510,895
**Ref**	C	T	C	T	A	G
**Alt**	A	C	T	C	G	A
**rsID**	rs3738476	rs17261772	rs26722	rs301	rs2720207	rs2240202
**AAF**						
**El Hierro**	0.904	0.572	0.091	0.197	0.288	0.135
**La Palma**	0.864	0.495	0.103	0.239	0.304	0.185
**La Gomera**	0.883	0.635	0.120	0.222	0.301	0.135
**Tenerife**	0.851	0.540	0.168	0.264	0.276	0.143
**Gran Canaria**	0.880	0.512	0.082	0.274	0.363	0.137
**Fuerteventura**	0.924	0.554	0.130	0.152	0.326	0.130
**Lanzarote**	0.828	0.613	0.113	0.216	0.309	0.132

AAF, alternative allele frequency; Alt, alternative allele; Chr, chromosome; Ref, reference allele.

Table S14. Population Branch Statistic (PBS) for six prioritized variants with similar alternative allele frequencies (AAF) between Canary Islanders and the North African (NAF) population, but different from the European (EUR) populations.

Chromosome	Position	rsID	PBS
1	151,006,539	rs3738476	0.303
2	135,911,422	rs17261772	0.107
5	33,963,870	rs26722	0.025
8	19,816,934	rs301	0.133
14	102,349,907	rs2720207	0.020
15	28,510,895	rs2240202	0.003

EUR includes GBR (British from England and Scotland), CEU (Utah Residents with Northern and Western European ancestry), and IBS (Iberian population in Spain) from 1KGP.

Table S15. Pairwise genetic distances (F_{ST}) between Canary Islands, North African (NAF), and different European (EUR) populations in the six prioritized variants with similar alternative allele frequencies (AAF) between Canary Islanders and North African population, but differing from the European populations.

Variant			F_{ST} Canary Islands *vs.*				F_{ST} NAF *vs.*		
**Chr**	**Position**	**rsID**	**CEU**	**GBR**	**IBS**	**NAF**	**CEU**	**GBR**	**IBS**
1	151,006,539	rs3738476	0.208	0.142	0.180	0.023	0.337	0.265	0.306
2	135,911,422	rs17261772	0.214	0.169	0.063	0	0.206	0.161	0.057
5	33,963,870	rs26722	0.096	0.072	0	0.009	0.166	0.141	0.033
8	19,816,934	rs301	0.048	0.124	0.055	0.013	0.133	0.230	0.142
14	102,349,907	rs2720207	0.015	0.105	0.047	0	0.015	0.105	0.046
15	28,510,895	rs2240202	0	0.093	0.003	0.008	0.036	0.160	0

CEU, Utah Residents with Northern and Western European ancestry (1KGP); Chr, chromosome; GBR, British from England and Scotland (1KGP); IBS, Iberian population in Spain (1KGP); NAF, North African population.

Supplemental figures

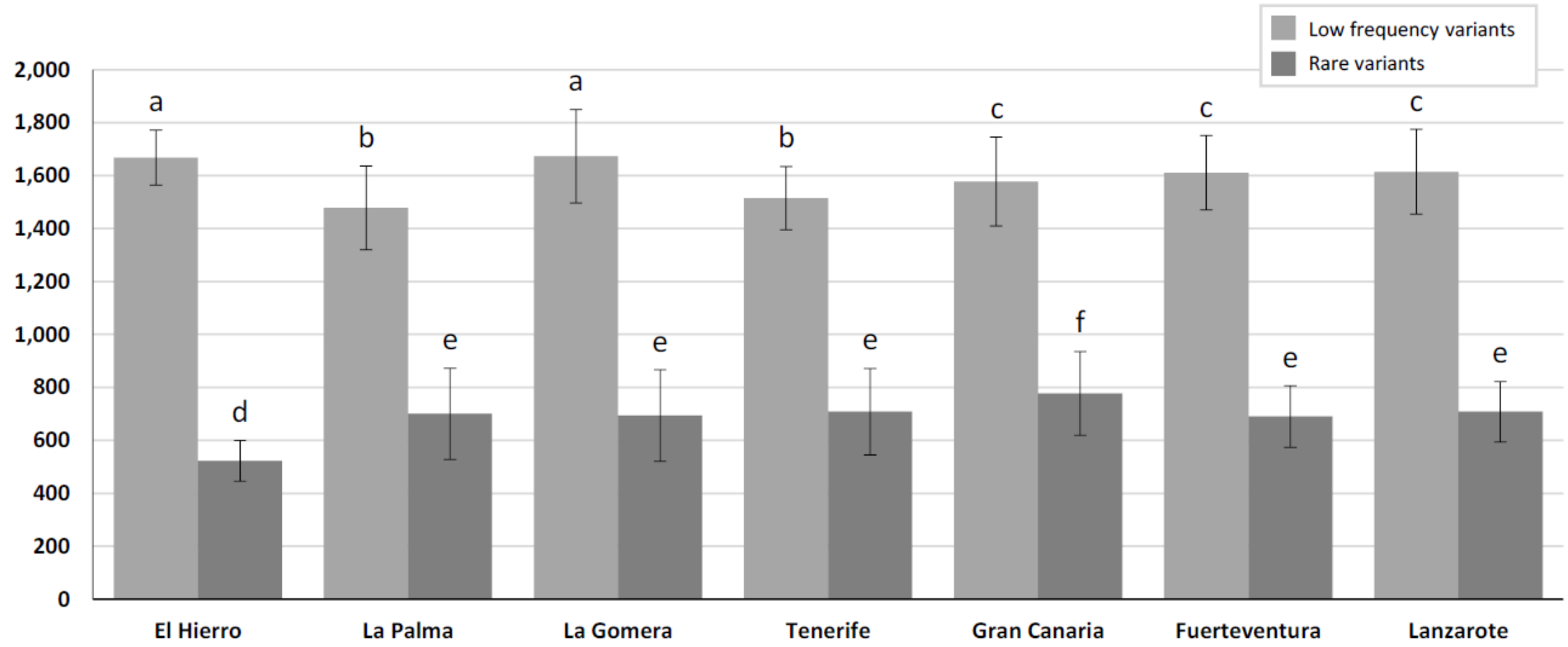

Figure S1. Mean number of variants per island, per individual (± SD) within the two lowest allele frequency ranges. Letters indicate the results of Duncan's multiple range test ($p<0.05$): islands sharing a letter show non-significant differences, whereas islands lacking letters in common show statistically significant differences. Bars represent average ± SD.

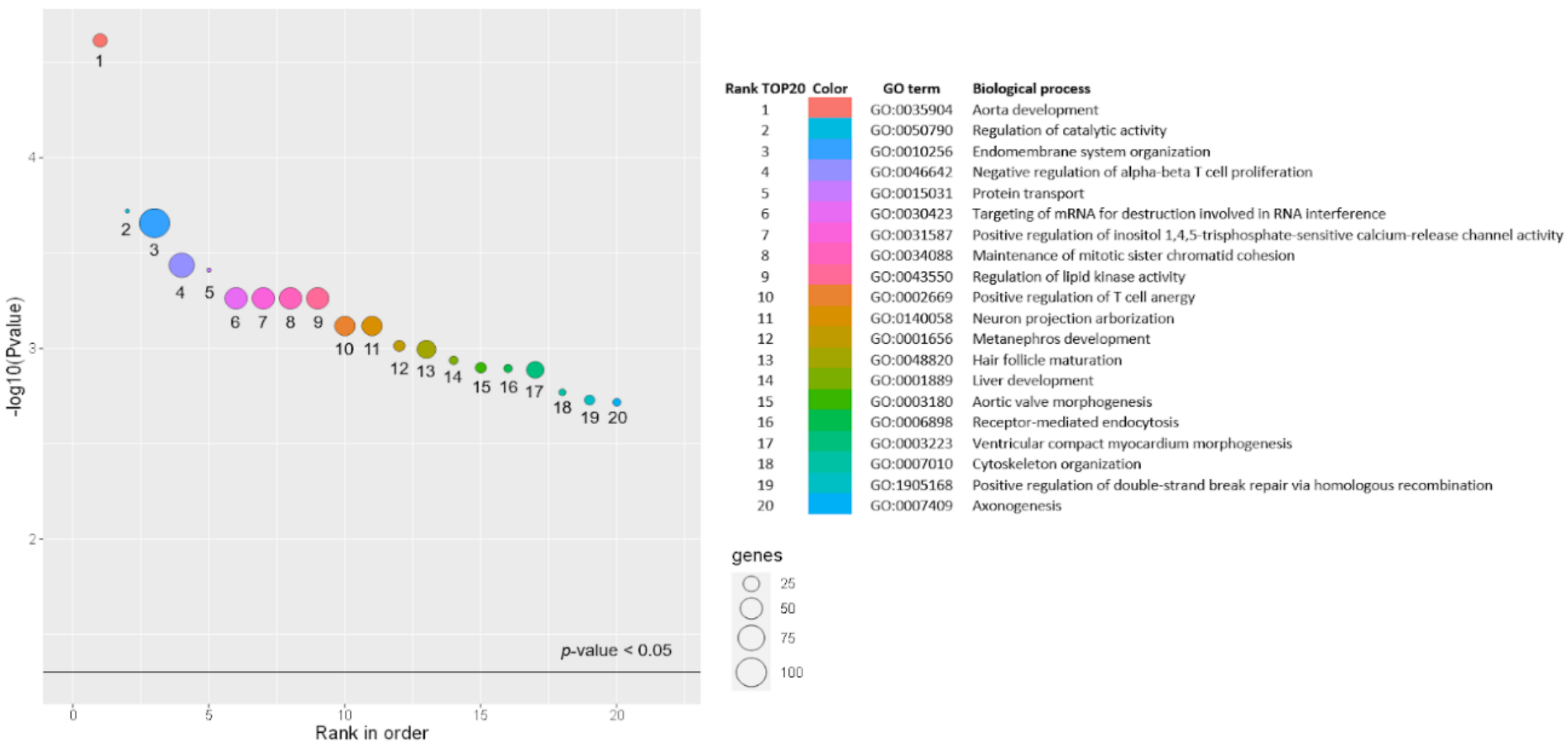

Figure S2. Enrichment analysis of the 123 genes containing variants above the mean (> two variants) that meet the criteria for HC LoF, C-score >MSC, and pLI >0.9. The size of the bubbles represents the percentage of functional genes covered by the user-defined gene list.

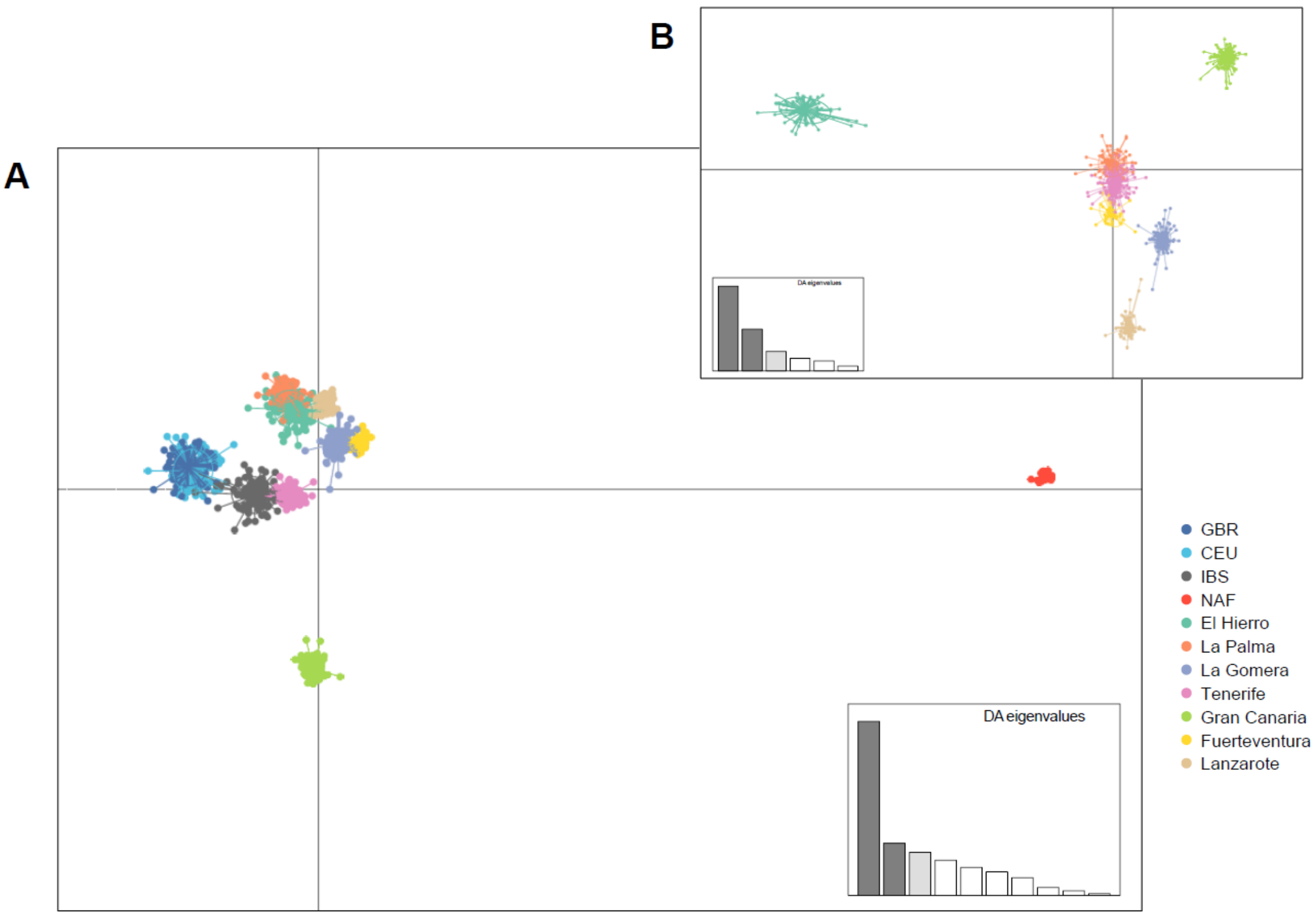

Figure S3. Discriminant Analysis of Principal Components (DAPC) for rare variants. A.) Including Canary Islands and reference populations, excluding outlier populations (SSA and FIN). **B.)** Considering only Canary Islanders. LD1, horizontal axis; LD2, vertical axis. CEU, Utah Residents with Northern and Western European ancestry (1KGP); GBR, British (1KGP); IBS, Iberian population in Spain (1KGP); NAF, North African population.

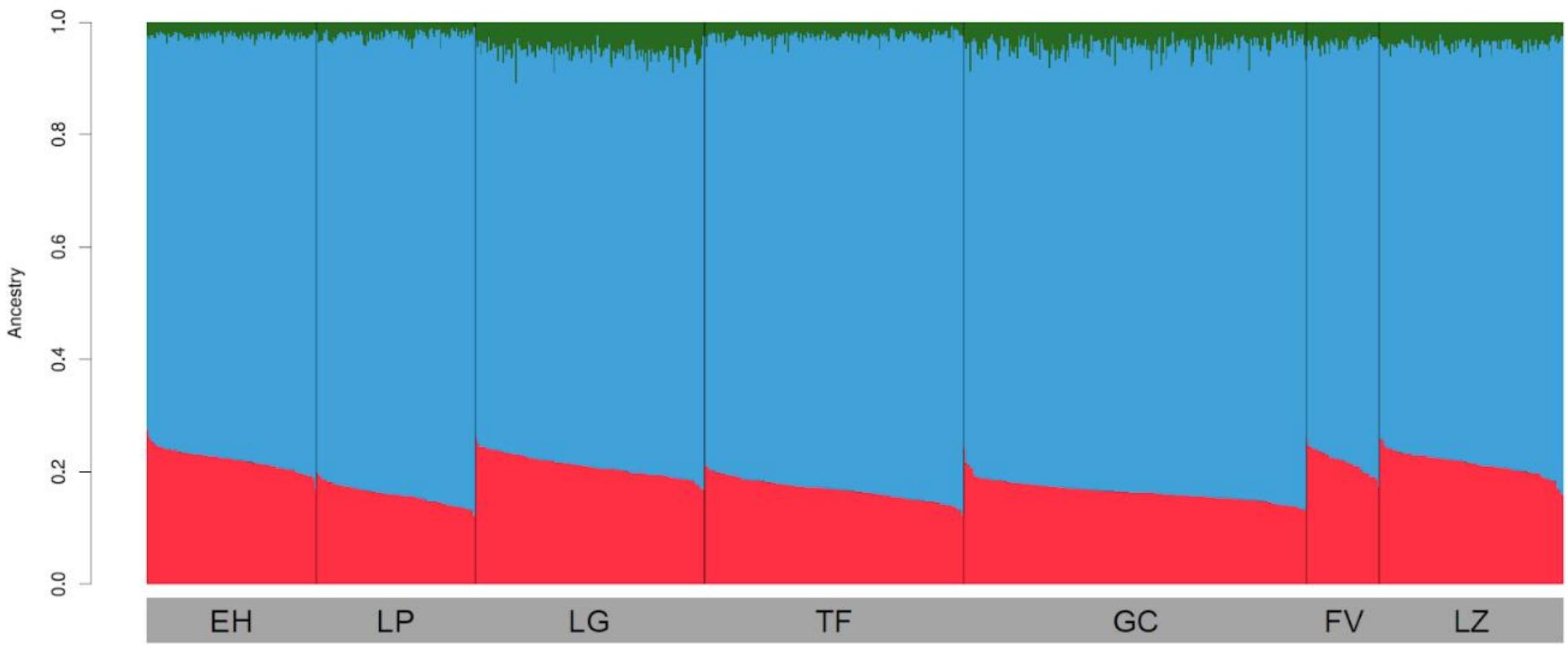

Figure S4. Global ancestry estimates per island, per individual, based on a three-way admixture model of European (blue), North African (red), and sub-Saharan African (green) populations, as provided by ELAI. From left to right: El Hierro (EH), La Palma (LP), La Gomera (LG), Tenerife (TF), Gran Canaria (GC), Fuerteventura (FV), and Lanzarote (LZ).

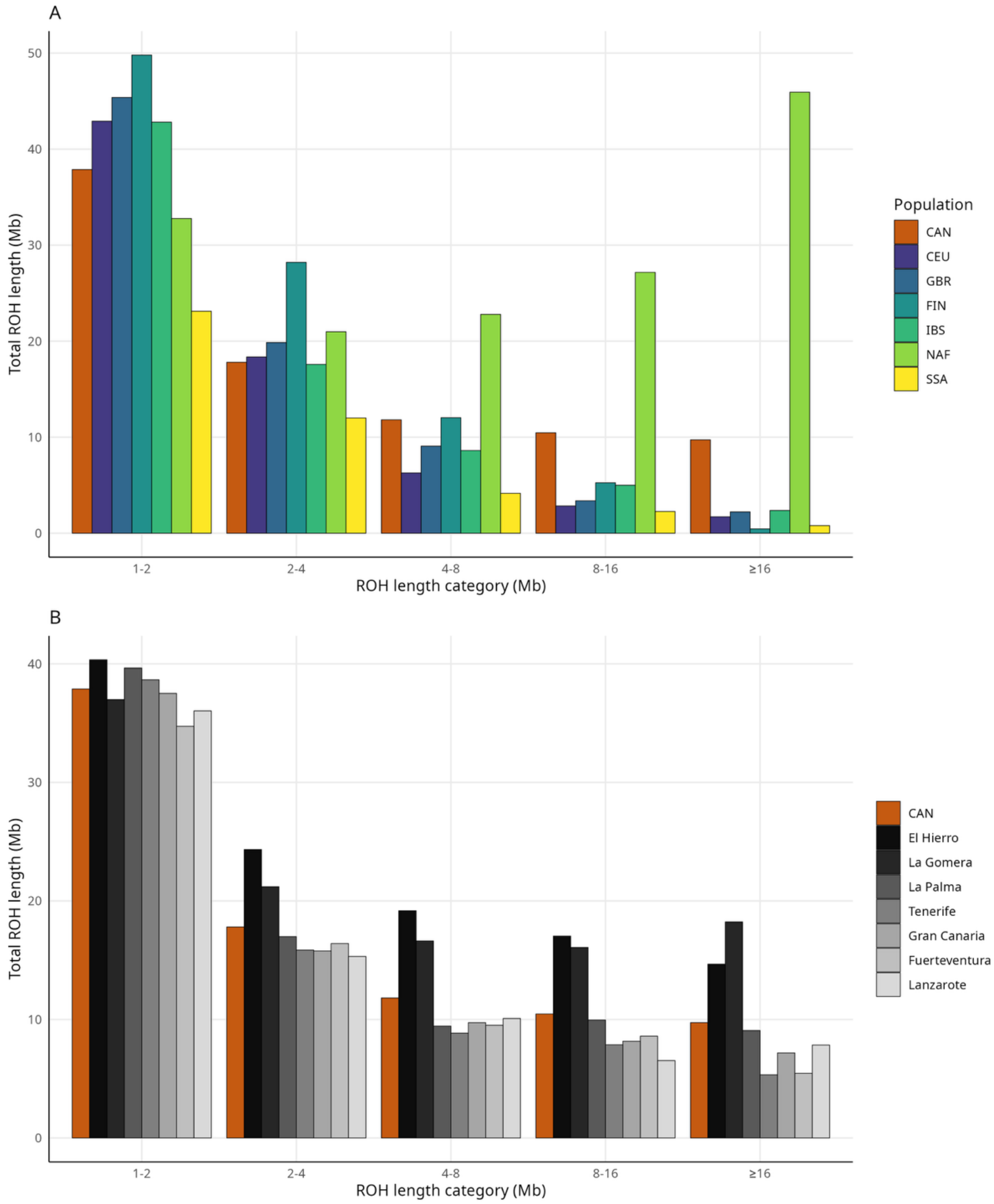

Figure S5. Distribution of runs of homozygosity (ROHs) across ROH length classifications (1-2, 2-4, 4-8, 8-16, and ≥16 Mb). A.) Reference datasets compared to the Canary Islands. **B.)** The Canary Islands. CAN, Canary Islands in aggregate; CEU, Utah Residents with Northern and Western European ancestry (1KGP); GBR, British (1KGP); FIN, Finnish (1KGP); IBS, Iberian population in Spain (1KGP); NAF, North African population; SSA, Yoruba population in Ibadan (1KGP).

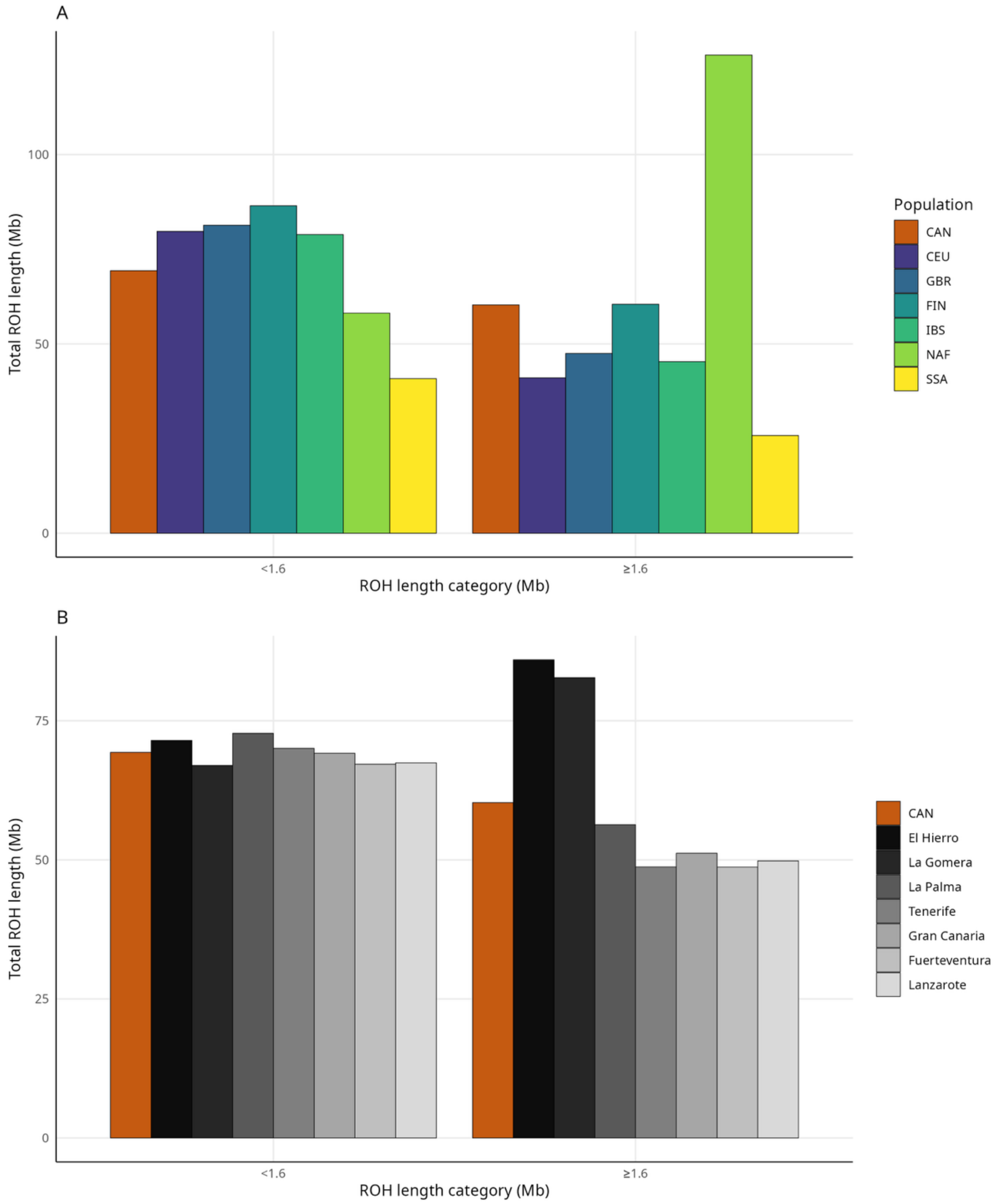

Figure S6. Distribution of runs of homozygosity (ROHs) based on the modified Pemberton classification (<1.6 Mb and ≥ 1.6 Mb) by average length (Mb). A.) Reference datasets compared to the Canary Islands. **B.)** The Canary Islands. CAN, Canary Islands in aggregate; CEU, Utah Residents with Northern and Western European ancestry (1KGP); GBR, British (1KGP); FIN, Finnish (1KGP); IBS, Iberian population in Spain (1KGP); NAF, North African population; SSA, Yoruba population in Ibadan (1KGP).

Supplemental references

S1. Nicolazzi, E.L., Iamartino, D., and Williams, J.L. (2014). AffyPipe: an open-source pipeline for Affymetrix Axiom genotyping workflow. Bioinformatics *30*, 3118–3119. https://doi.org/10.1093/bioinformatics/btu486.

S2. Guillen-Guio, B., Lorenzo-Salazar, J.M., González-Montelongo, R., Díaz-de Usera, A., Marcelino-Rodríguez, I., Corrales, A., Cabrera de León, A., Alonso, S., and Flores, C. (2018). Genomic analyses of human European diversity at the southwestern edge: Isolation, African influence and disease associations in the canary islands. Mol. Biol. Evol. *35*, 3010–3026. https://doi.org/10.1093/molbev/msy190.

S3. Serradell, J.M., Lorenzo-Salazar, J.M., Flores, C., Lao, O., and Comas, D. (2024). Modelling the demographic history of human North African genomes points to a recent soft split divergence between populations. Genome Biol. *25*, 201. https://doi.org/10.1186/s13059-024-03341-4.

S4. Danecek, P., Bonfield, J.K., Liddle, J., Marshall, J., Ohan, V., Pollard, M.O., Whitwham, A., Keane, T., McCarthy, S.A., Davies, R.M., et al. (2021). Twelve years of SAMtools and BCFtools. Gigascience *10*. https://doi.org/10.1093/gigascience/giab008.

S5. Freeman, T.M., Genomics England Research Consortium, Wang, D., and Harris, J. (2020). Genomic loci susceptible to systematic sequencing bias in clinical whole genomes. Genome Res. *30*, 415–426. https://doi.org/10.1101/gr.255349.119.

S6. Karczewski, K.J., Francioli, L.C., Tiao, G., Cummings, B.B., Alföldi, J., Wang, Q., Collins, R.L., Laricchia, K.M., Ganna, A., Birnbaum, D.P., et al. (2020). The mutational constraint spectrum quantified from variation in 141,456 humans. Nature *581*, 434–443. https://doi.org/10.1038/s41586-020-2308-7.

S7. Li, H., and Durbin, R. (2009). Fast and accurate short read alignment with Burrows-Wheeler transform. Bioinformatics *25*, 1754–1760. https://doi.org/10.1093/bioinformatics/btp324.

S8. O’Connor, B.D., and van der Auwera, G. (2020). Genomics analysis with Spark, Docker and clouds (O’Reilly Media).

S9. Wienbrandt, Lars, Christoph Prieß, Jan Christian Kässens, Andre Franke, Franziska Uhing, and David Ellinghaus (2022). EagleImp-Web: A Fast and Secure Genotype Phasing and Imputation Web Service Using Field-Programmable Gate Arrays. bioRxiv, 2022.02.24.481790. https://doi.org/10.1101/2022.02.24.481790.

S10. Ghoussaini, M., Mountjoy, E., Carmona, M., Peat, G., Schmidt, E.M., Hercules, A., Fumis, L., Miranda, A., Carvalho-Silva, D., Buniello, A., et al. (2021). Open Targets Genetics: systematic identification of trait-associated genes using large-scale genetics and functional genomics. Nucleic Acids Res. *49*, D1311–D1320. https://doi.org/10.1093/nar/gkaa840.

S11. Ségurel, L., and Bon, C. (2017). On the evolution of lactase persistence in humans. Annu. Rev. Genomics Hum. Genet. *18*, 297–319. https://doi.org/10.1146/annurev-genom-091416-035340.

S12. Bitarello, B.D., de Filippo, C., Teixeira, J.C., Schmidt, J.M., Kleinert, P., Meyer, D., and Andrés, A.M. (2018). Signatures of long-term balancing selection in human genomes. Genome Biol. Evol. *10*, 939–955. https://doi.org/10.1093/gbe/evy054.

S13. DeGiorgio, M., Lohmueller, K.E., and Nielsen, R. (2014). A model-based approach for identifying signatures of ancient balancing selection in genetic data. PLoS Genet. *10*, e1004561. https://doi.org/10.1371/journal.pgen.1004561.

S14. de Bakker, P.I.W., McVean, G., Sabeti, P.C., Miretti, M.M., Green, T., Marchini, J., Ke, X., Monsuur, A.J., Whittaker, P., Delgado, M., et al. (2006). A high-resolution HLA and SNP haplotype map for disease association studies in the extended human MHC. Nat. Genet. *38*, 1166–1172. https://doi.org/10.1038/ng1885.

S15. Lindo, J., Huerta-Sánchez, E., Nakagome, S., Rasmussen, M., Petzelt, B., Mitchell, J., Cybulski, J.S., Willerslev, E., DeGiorgio, M., and Malhi, R.S. (2016). A time transect of exomes from a Native American population before and after European contact. Nat. Commun. *7*, 13175. https://doi.org/10.1038/ncomms13175.
